# Single-cell profiling of the microenvironment in human bone metastatic renal cell carcinoma

**DOI:** 10.1038/s42003-024-05772-y

**Published:** 2024-01-12

**Authors:** Fen Ma, Shuoer Wang, Lun Xu, Wending Huang, Guohai Shi, Zhengwang Sun, Weiluo Cai, Zhiqiang Wu, Yiming Huang, Juan Meng, Yining Sun, Meng Fang, Mo Cheng, Yingzheng Ji, Tu Hu, Yunkui Zhang, Bingxin Gu, Jiwei Zhang, Shaoli Song, Yidi Sun, Wangjun Yan

**Affiliations:** 1 Shanghai Key Laboratory of Compound Chinese Medicines, The MOE Key Laboratory for Standardization of Chinese Medicines, Institute of Chinese Materia Medica, Shanghai University of Traditional Chinese Medicine, 1200 Cailun Road, 201203 Shanghai, China; 2 Institute of Neuroscience, CAS Center for Excellence in Brain Science and Intelligence Technology, Chinese Academy of Sciences, 320 Yueyang Road, Shanghai, China; 3 Department of Musculoskeletal Surgery, Fudan University Shanghai Cancer Center, 270 Dong’an Road, Shanghai, China; 4 Department of Nuclear Medicine, Fudan University Shanghai Cancer Center, 270 Dong’an Road, Shanghai, China; 5 Department of Oncology, Shanghai Medical College, Fudan University, 138 Medical College Road, Shanghai, China; 6 Department of Urology, Fudan University Shanghai Cancer Center, 270 Dong’an Road, Shanghai, China; 7 Department of Orthopedic, Naval Medical Center of PLA, Second Military Medical University, 338 Huaihai West Road, Shanghai, China; 8 Department of Anesthesiology, Fudan University Shanghai Cancer Center, 270 Dong’an Road, Shanghai, China

**Keywords:** Cancer microenvironment, Bone metastases, Cancer microenvironment

## Abstract

Bone metastasis is of common occurrence in renal cell carcinoma with poor prognosis, but no optimal treatment approach has been established for bone metastatic renal cell carcinoma. To explore the potential therapeutic targets for bone metastatic renal cell carcinoma, we profile single cell transcriptomes of 6 primary renal cell carcinoma and 9 bone metastatic renal cell carcinoma. We also include scRNA-seq data of early-stage renal cell carcinoma, late-stage renal cell carcinoma, normal kidneys and healthy bone marrow samples in the study to better understand the bone metastasis niche. The molecular properties and dynamic changes of major cell lineages in bone metastatic environment of renal cell carcinoma are characterized. Bone metastatic renal cell carcinoma is associated with multifaceted immune deficiency together with cancer-associated fibroblasts, specifically appearance of macrophages exhibiting malignant and pro-angiogenic features. We also reveal the dominance of immune inhibitory T cells in the bone metastatic renal cell carcinoma which can be partially restored by the treatment. Trajectory analysis showes that myeloid-derived suppressor cells are progenitors of macrophages in the bone metastatic renal cell carcinoma while monocytes are their progenitors in primary tumors and healthy bone marrows. Additionally, the infiltration of immune inhibitory *CD47*^+^ T cells is observed in bone metastatic tumors, which may be a result of reduced phagocytosis by *SIRPA*-expressing macrophages in the bone microenvironment. Together, our results provide a systematic view of various cell types in bone metastatic renal cell carcinoma and suggest avenues for therapeutic solutions.

## Abstract (web summary)

Single-cell profiling of primary and bone metastatic renal cell carcinoma suggests variation in intratumoral heterogeneity between samples and the is a valuable resource for examining the underlying pathogenic mechanisms for these cancers.

## Introduction

Renal cell carcinoma (RCC) is one of the most malignant tumors worldwide, with ~400,000 new cases and almost 200,000 deaths annually^1^. In the United States, 76,080 individuals were diagnosed as RCC in 2021, accounting for 4% of newly occurrent malignant tumors and 46.4% of urinary tumors^2^. Eighty-five percent of the pathological diagnosis for RCC is clear cell renal cell carcinoma (ccRCC)^3^, consistent with the putative cell of origin for RCC using scRNA-seq analyses^4^. It is worth noting that the ccRCC showed a high risk of distant organ metastasis, and the five-year survival rate of RCC patients would significantly decrease from 93% to 12% when distant metastasis occurred^5^. More than one-third of metastatic RCC patients were accompanied by bone metastases^6^.

Bone metastatic renal cell carcinoma (BMRCC) patients are usually complicated by skeletal related events including pathological fractures, spinal cord compression, and hypercalcemia^7,8^. Until now, the main treatment options for BMRCC in clinical application are extensive surgical resection and radiotherapy^9,10^. Approved bone-targeted systemic therapies like bisphosphonates and denosumab showed limited benefits to the improvement of overall survival^11^. Although certain target-based agents such as antiangiogenic therapy have shown promising effectiveness, the progression-free survival of BMRCC remains low of 4.7 months versus 11.2 months for those without bone metastases^12^. Therefore, systematic molecular characterization of BMRCC by single-cell transcriptome data may help discover predictive biomarkers and identify therapeutic targets for improvement of BMRCC treatment.

The intrinsic genetic heterogeneity and dynamic immunogenic features significantly affect the therapeutic outcomes. Previous studies have explored in-depth tumor microenvironment profiling of ccRCC at single-cell level^13^. Tumor epithelial cells of ccRCC have been reported to play an active role in promoting immune cell infiltration^4^, while high proportions of endothelial cells were associated with lack of response to immunotherapy in ccRCC^4^. The CD8^+^ T cells and macrophages were reported to be increased in the tumor micro-environment^14^. The cytotoxic T cell subsets expressed higher levels of co-inhibitory receptors and effector molecules in RCC patients with effective response to immune checkpoint blockade^15^, and the maintenance of expanded T cell clones were correlated with drug response to anti-PD-1 therapy^16^. In addition to the classical roles of phagocytosis and antigen presentation, myeloid cells could impact response to cancer therapy^17^ and directly contribute to tumor progression and metastases^18^. Besides, macrophages in RCC with effective responses to immune checkpoint blockade exhibited pro-inflammatory characteristic^15^. Similarly, *TREM2*/*APOE*/*C1Q*-positive macrophage was identified as a potential prognostic biomarker for ccRCC recurrence by single-cell protein analysis^19^. In addition, exhausted CD8^+^ T cells and M2-like macrophages showed co-occurrence in advanced ccRCC and expressed ligands and receptors supporting T cell dysfunction and M2-like polarization^20^. As a primary hematopoietic organ, bone marrow represents a unique reservoir for several types of immune cells, which would dramatically influence the trajectory of malignant disease. However, our incomplete understanding of the tumor microenvironment and heterogeneity of BMRCC hinder the efficient translation of these findings iBMBnto therapeutic treatment.

Although important insights have been drawn for bone metastases treatment in the past decades, there remain multiple longitudinal barriers to gain a better understanding of the cell compositions and interconnections in the bone metastatic microenvironment. Traditional bulk transcriptome investigation is limited by insufficient resolution to characterize specific cellular types and expression of ligands and receptors of diverse cell types due to the average measuring of cell populations. Here, we systematically collect both primary and bone metastatic tumor tissues from ccRCC and performed scRNA-seq to explore the ecosystem of tumor, immune and stromal cells. The current study will provide additional therapeutic targets given a deeper insight into the cellular and molecular characteristics of BMRCC.

## Results

### Cell landscape of primary and bone metastatic renal cell carcinoma

**Fig. 1:**
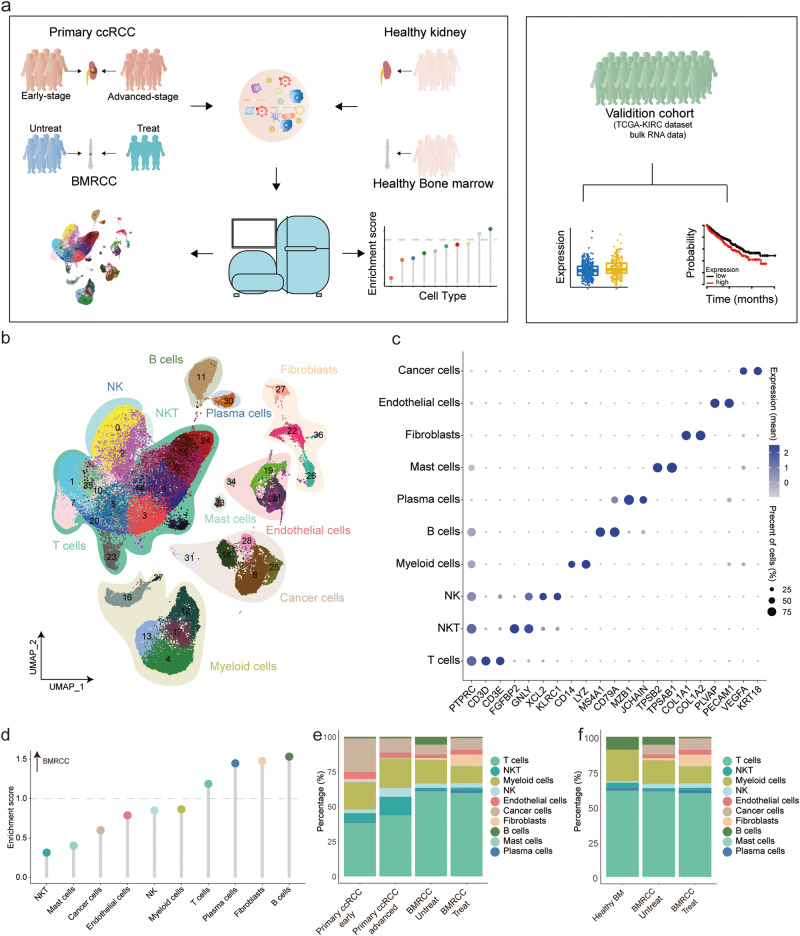
The major cell clusters revealed by the primary ccRCC and BMRCC. **a** Workflow showing the process of sample collection, single-cell dissociation, sorting, sequencing, and data analysis. **b** UMAP plot of all single cells from the primary ccRCC of early and advanced stages and BMRCC with or without treatment. Cell colors indicate unsupervised clustering subgroups. Shading ranges and colors indicate cell type. **c** Bubble heatmap displaying the expression levels of representative well-known markers across the cell types identified in the cohort. Dot size indicates fraction of cells with expression of the indicated gene, and colors represent the normalized expression levels. **d** Lollipop plot showing the tissue distribution of each major cell type by Ro/e analysis in the BMRCC compared with the primary ccRCC. Dot color indicate the cell type labeled. BMRCC-enriched types were characterized with Ro/e > 1. Fisher’s exact test was used to compare significance. **e** The major cell type proportions among the primary ccRCC of early and advanced stages and BMRCC with or without treatment. Colors on the columns indicate cell type. **f** The major cell type proportions in the heathy bone marrows and BMRCC with or without treatment. Colors on the columns indicate cell type.

To explore the cellular and molecular basis of bone metastasis of renal cell carcinoma, we collected 6 primary and 9 bone metastatic tumors from 14 ccRCC patients for scRNA-seq analyses (Fig. 1a, Supplementary Fig. 1a, b and Supplementary Table 1). Among them, 3 BMRCC patients were treated with tyrosine kinase inhibitor (TKI) and PD-1 inhibitor (Supplementary Table 1). In addition, single-cell RNA-seq samples of 6 primary ccRCC in different stages, 6 healthy kidneys and 6 healthy bone marrows were enrolled in the study from public datasets^21–24^ for elucidating the unique characteristics of BMRCC. In total, we obtained single cell transcriptomes from a total of 258,084 cells. After stringent quality control, 33,119 cells from early primary tumors, 27,275 cells from advanced primary tumor, 64,582 cells from bone metastatic tumors (Supplementary Fig. 2a and Supplementary Table 2), 27,210 cells from healthy kidney, and 18,396 cells from healthy bone marrows were reserved. Integration of all the cells using unsupervised graph-based clustering revealed 11 major cell types (Fig. 1b and Supplementary Fig. 2b, c), which were further annotated based on canonical cell markers. Specifically, the immune cell types consisted of T cells (*CD3D* and *CD3E*), NKT cells (*GNLY* and *FGFBP2*), NK cells (*XCL2* and *KLRC1*), myeloid cells (*CD14*, *FCGR3A*, and *LYZ*), B cells (*CD79A* and *MS4A1*), mast cells (*TPSB1* and *TPSAB1*), and plasma cells (*MZB1* and *JCHAIN*). The non-immune cells included endothelial cells (*PVALP* and *PECAM1*), CAFs (*COL1A1* and *COL1A2*), and cancer cells (*KRT18* and *VEGFA*) (Fig. 1c).

We next compared the relationship between the major cell types and BMRCC, with primary ccRCC and healthy bone marrows as references. We calculated an enrichment score using Ro/e analysis which compared the ratio of each cell type in BMRCC with that in primary ccRCC or healthy bone marrows (Fig. 1d and Supplementary Fig. 2d–f). The results showed that B cells, plasma cells and T cells were enriched in BMRCC tumors (Fig. 1d, e and Supplementary Fig. 2d), indicating the infiltration of lymphoid cells in the bone metastasis microenvironment. By contrast, NKT cells, mast cells and cancer cells were enriched in early- and late-stage primary ccRCC (Fig. 1d, e). Mast cells in tumors were thought to play a dual role in influencing the fate of tumor cells^25^, the enrichment of mast cells in primary ccRCC instead of BMRCC hinted that mast cells might exhibit diverse functions across different tumor environment. Interestingly, the treatment-naïve BMRCC patients showed increased infiltration of B cells and myeloid cells in comparison with the BMRCC patients with the immunotherapy treatment (Fig. 1e, f, and Supplementary Fig. 2e), suggesting that the immunotherapy treatment might help to reshape the immune microenvironment of BMRCC.

### Tumor cells in bone metastasis have stronger angiogenesis ability

**Fig. 2:**
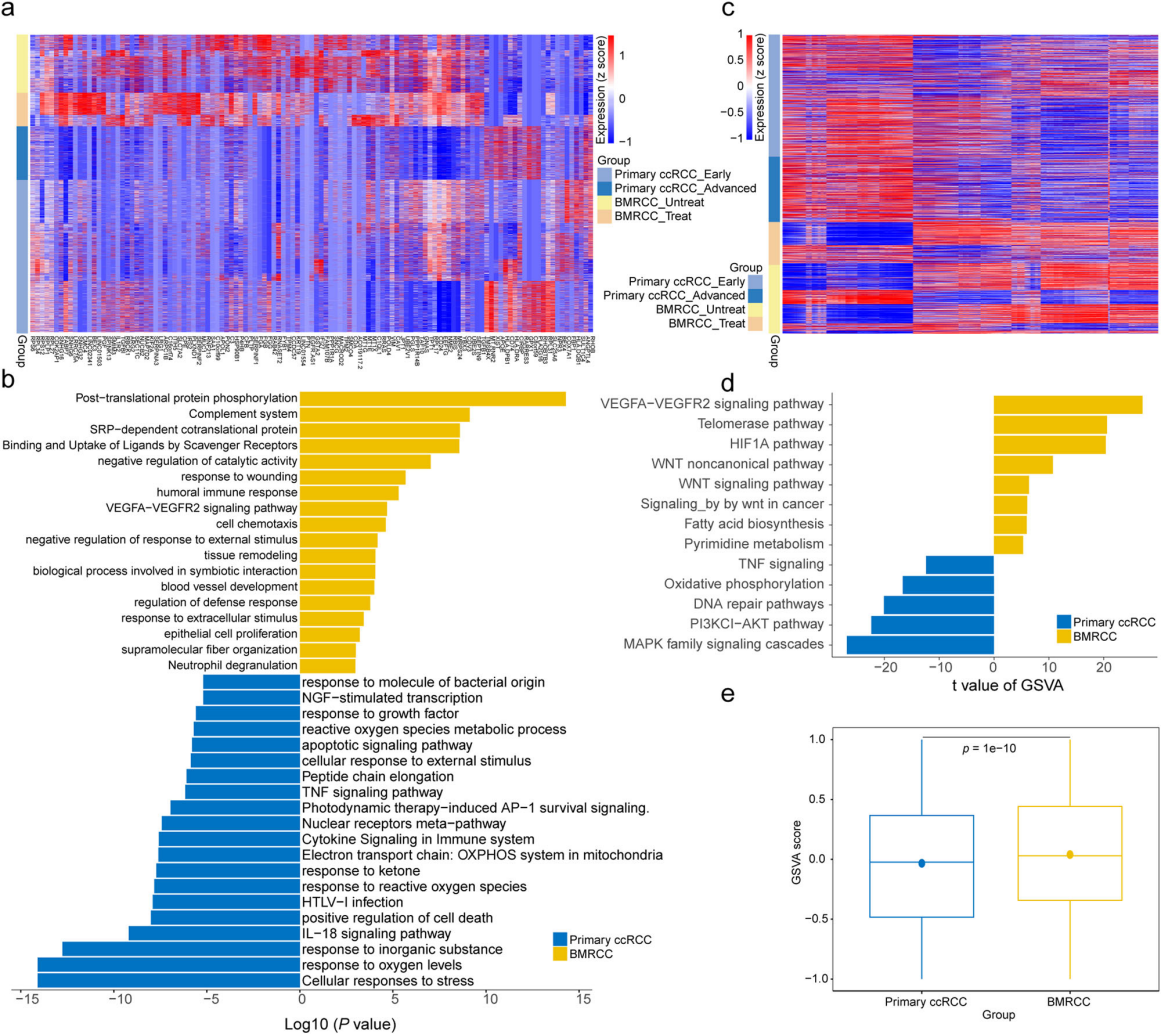
Cancer cell profiles in the primary ccRCC and BMRCC. **a** Heatmap showing the differentially expressed genes between primary ccRCC and BMRCCs. The single cells are ordered by their tissue origins marked by the legend column on the left. Color from red to blue indicates a high to low gene expression. **b** Bar plot showing functional enrichment of genes highly expressed in the primary ccRCC or BMRCC. The yellow columns indicate BMRCC group. The blue columns indicate primary ccRCC group. **c** Heatmap showing the copy number variations score of genes located in the chromosome 8. The cells are ordered by their tissue origins marked by the legend column on the left. Color from red to blue indicates a high to low gene expression. **d** Bar plot showing the enriched pathways of genes located on the chr8 amplified region by GSVA. The yellow columns indicate BMRCC group. The blue columns indicate primary ccRCC group. The *x*-axis showed t values calculated by limma regression. **e** Comparison of GSVA scores of the *WNT* signaling pathway between the primary ccRCC and BMRCC. Yellow columns indicate BMRCC group, blue columns indicate primary ccRCC group. Centre line indicates median, box represents first and third quantiles, and whiskers indicate maximum and minimum values. *P* value was calculated by Student’s *t* test, *n* = 11132 biologically independent cells. Effect size of Cohen’s d: 0.135.

We next dissected the gene signatures of all tumor cells in the cohort and found that the primary and bone metastatic enriched cancer cells revealed distinct gene signatures (Fig. 2a and Supplementary Fig. 3a–c). Notably, genes with higher expression levels in the bone metastatic tumors demonstrated a pro-angiogenic signature, and were enriched in blood vessel development and *VEGFA*-*VEGFR2* signaling pathways (Fig. 2a, b and Supplementary Fig. 3a, b), suggesting the angiogenesis ability of cancer cells in the BMRCC. By contrast, subpopulations in the primary tumors exhibited higher expression of genes associated with response to oxygen levels, *IL-18* signaling pathway and *TNF* signaling pathway (Fig. 2b). In addition, higher MHC associated genes (e.g., *HLA-B* and *HLA-C*) were highly expressed in primary ccRCC (Supplementary Fig. 3c). major histocompatibility complex class II is critical for antigen presentation to T cells, and is important for the efficacy of immunotherapy^26^. We also observed that the BMRCC patients treated with immunotherapy showed downregulation of *TP53*-regulated genes (e.g., *TP53I3*, *COX6C* and *TPM2*) (Supplementary Fig. 3d).

To further investigate genetic heterogeneity between tumor cells in primary and bone metastatic tumors, we inferred copy-number alterations for all the tumor cells, with the fibroblasts and endothelial cells as normal ploidy controls (Supplementary Fig. 3e). We found aberrant copy-number alterations regions in the short arm of chromosome 3, long arm of chr13, and chr14. Specifically, extensive chromosomal gains were observed in the long arm of chr5 and chr16. Of note, deletions in chromosome 3p tended to be a universal truncal event in ccRCC, as this region contains the *VHL* tumor suppressor locus^27,28^. TCGA-KIRC cohort confirmed that *VHL* gene was the most common mutation in ccRCC patients (Supplementary Fig. 3f). Interestingly, we also observed a copy number amplification on chromosome 8q in the bone metastatic samples (*P* < 2.2e−16, Student’s *t* test) (Fig. 2c and Supplementary Fig. 3e). Functional analysis of genes located in the 8q amplification region showed a significant enrichment in *WNT* signaling pathway (Fig. 2d), an ancient and evolutionarily conserved pathway that regulates crucial aspects of cell fate determination and cell migration. Further examination showed that the activation of *WNT* signaling pathway in the BMRCC tumors was significantly higher than that of primary ccRCC tumors (Fig. 2e). These results suggested the important regulation roles of angiogenesis and *WNT* signaling pathway in the BMRCC cells and provided a potential therapeutic solution to targeting their activities in clinical treatment.

### Cancer-associated fibroblasts were associated with metastasis and poor prognosis of RCC

**Fig. 3:**
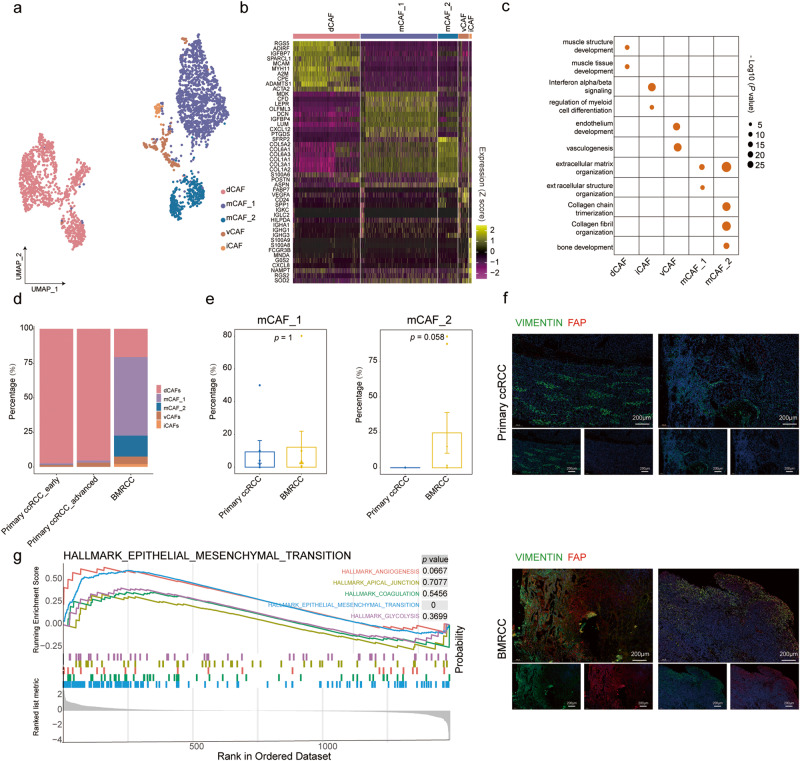
High proportion of mCAF cells in the BMRCC. **a** UMAP plot showing the subtyping of CAFs. Colors of the cell indicate the cell type marked by legend. **b** Heatmap showing the expression levels of top 10 marker genes for CAF subtypes. The single cells are ordered by cell types marked by the upper legend column. Color from yellow to purple indicates a high to low gene expression. **c** Dot plot showing the functionally enriched pathways of marker genes in each CAF subtype. Dot color and size indicate *p* value. **d** Bar plot showing the fraction of CAF subtypes in the primary ccRCC and BMRCC. Colors on the columns indicate cell type. **e** Comparison of the fraction of mCAF_1 and mCAF_2 cells among the primary ccRCC and BMRCC. Yellow columns indicate BMRCC group, blue columns indicate primary ccRCC group. Data presents the mean ± SEM. *P* values were calculated by wilcox rank sum test, *n* = 15 biologically independent samples. The effect size of Cohen’s d: mCAF_1: 0.118; mCAF_2: 0.822. **f** Immunostaining experiments showing the existence of mCAF_2 in BMRCC and primary ccRCC. The green color indicates the expression of VIMENTIN protein. The red color indicates the expression of FAP protein. **g** GSEA analysis showing the enrichment of top 5 ranked hallmark pathways between mCAF_1 and mCAF_2 subtypes. The colors of the line indicate the pathway of enrichment.

As the most prevalent component in the tumor microenvironment, CAFs play diverse roles in driving tumorigenesis and affecting response to treatment^29^. Thus, we next compared the heterogeneity of CAFs between primary ccRCC and BMRCCs. Focused examination of the CAF compartment revealed 5 subclusters based on canonical cell markers (Fig. 3a, b). Common fibroblast marker genes such as *S100A4*, *SPARCL1* and non-specific mesenchymal markers *VIM* and *SPARC* were found to be expressed across all subgroups (Supplementary Fig. 4a). Gene ontology (GO) analysis using marker gene signatures in each subpopulation showed differential preferences for functional pathways (Fig. 3c). Specifically, developmental CAFs (dCAFs), with high expression of *MYH11* and *MCAM*, was functionally featured by muscle structure and tissue development (Fig. 3b, c and Supplementary Fig. 4b), and presented in both primary ccRCC and BMRCC (Fig. 3d and Supplementary Fig. 4c). While, inflammatory CAFs (iCAFs) was characterized by interferon alpha/beta signaling, regulation of myeloid cell differentiation, and major histocompatibility complex class I antigen presentation (Fig. 3c and Supplementary Fig. 4d), consistent with the characteristics of the recently described antigen-presenting CAF (apCAFs)^30^. Vascular CAFs (vCAFs) were featured by endothelium development and vasculogenesis (Fig. 3c).

Both matrix CAF (mCAF_1 and mCAF_2) clusters were enriched in the BMRCC patients (Fig. 3d, e), with high expression levels of extracellular matrix proteins (*COL5A2*, *AEBP1*, *COL1A1*, *COL1A2* and *COL3A1*) (Fig. 3c and Supplementary Fig. 4e). In addition to extracellular matrix organization, mCAF_2 was also enriched in collagen fibril organization with expression of *FAP*, *COMP*, *MMP13*, and *SFRP2* (Fig. 3c and Supplementary Fig. 4f). Given the role of CAFs in the assembly of fibronectin that is highly abundant in extracellular matrix and strongly associated with metastasis^31^, mCAF_2 might be the CAF subtype affecting bone metastasis of renal cell cancer. Comparative analysis also revealed that the mCAF_2 subtype was more abundant in BMRCC (Fig. 3d, e). The higher abundance of mCAF_2 in BMRCC than in primary ccRCC was validated by immunostaining experiments (Fig. 3f ). Besides, mCAF_2 exhibited significantly higher enrichment score than mCAF_1 in the epithelial mesenchymal transition (Fig. 3g), which is closely related to cancer progression and metastasis^32^. The proportion of mCAF_2 was not decreased in BMRCC samples with treatment, suggesting the little influence of fibroblasts upon immunotherapy. The expression of mCAF_2 marker genes was higher in ccRCC patients with stage III/IV (Supplementary Fig. 4g). We next grouped the patients with ccRCC into two groups based on the expression level of marker genes of mCAF_2, and found that the group with high expression of mCAF_2 markers showed significantly worse prognosis (Supplementary Fig. 4h). These results suggested that the mCAF_2 cluster is strongly associated with BMRCC and could predict poor prognosis for RCC patients.

### Diversity of T cell subtypes in BMRCCs

**Fig. 4:**
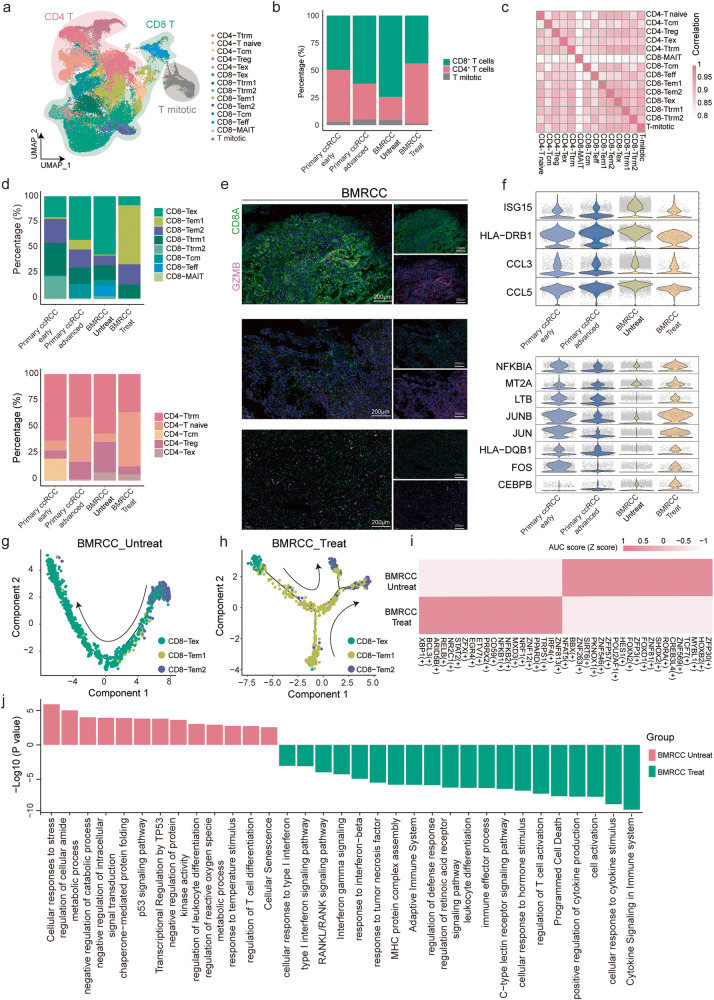
Inhibitory T cell subtypes were enriched in the microenvironment of BMRCC. **a** UMAP plot showing the 13 subtypes of T cells. The colors of the cell indicate the cell type marked by legend. **b** Bar plot showing the fractions of CD8^+^ T, CD4^+^ T and mitotic T cells in the primary ccRCC of early and advanced stages and BMRCC with or without treatment. Colors on the columns indicate cell type marked by legend. **c** Correlation heatmap showed the differences among T cell subtypes. Colors on heatmap indicate the correlation value. Color from pink to white indicates a high to low correlation. **d** The distribution of CD4^+^ T and CD8^+^ T subtypes among the primary ccRCC of early and advanced stages and BMRCC with or without treatment. Colors on the columns indicate cell type marked by legend. **e** Immunostaining experiments showing the existence of CD8-Tem in BMRCC. The green color indicates the expression of CD8 protein. The purple color indicates the expression of GZMB protein. **f** Violin plot showing the expression levels of indicated marker genes among the indicated groups. Colors on the columns indicate groups marked by *x*-axis. **g**, **h** Developmental trajectory of inhibitory T cell subtypes in the treatment-naïve (**g**) and treated (**h**) BMRCC. Colors of cell indicate the cell type markered by legend. **i** Differentially enriched TFs between the treatment-naïve and treated BMRCC. Colors on heatmap indicate TF AUC score of CD8-Tem1 in treatment-naïve and treated BMRCC group. Color from red to pink indicates a high to low AUC score. **j** Bar plot showing the enriched pathways of regulons of the TFs in CD8-Tem1 from the treatment-naïve and treated BMRCC. The red columns indicate BMRCC Untreat group. The green columns indicate BMRCC Treat group.

To reveal the intrinsic structure and potential functional subtypes of lymphoid cells, the T cell populations were further subdivided into 13 sub-clusters, including 5 clusters for CD4^+^ T cells, 8 clusters for CD8^+^ T cells and 1 mitotic T cell cluster (Fig. 4a, b and Supplementary Fig. 5a). Each of these clusters showed specific expression of unique signature genes (Fig. 4c). The proportions of CD8^+^ T cells were increased with tumor progression, and decreased in the BMRCC after treatment (Fig. 4b). Two clusters of inhibitor CD4^+^ T cells (CD4-Treg and CD4-Tex) were identified, CD4-Treg was characterized by specific expression of *FOXP3*, *TNFRSF9* and *TIGIT*, whereas CD4-Tex expressed high levels of *CTLA4*, *PDCD1* and *CXCL13* (Fig. 4a and Supplementary Fig. 5b). Both of them were dominant in bone metastatic tumors, and the proportion of CD4-Treg decreased in the BMRCC patients with treatment (Fig. 4d). We also found a large group of effect memory T cells (CD8-Tem) (Fig. 4a, e), which could be divided to two clusters (CD8-Tem1 and CD8-Tem2). CD8-Tem1 that had higher expression of *GZMB* and *CTSW* were enriched in bone metastatic tumors, whereas CD8-Tem2 cells were enriched in primary ccRCC with higher expression of *KLRD1*, *KLRF1*, and *KLRG1* (Fig. 4d and Supplementary Fig. 5c).

The proportion of exhausted T cell cluster CD8-Tex, with high expression levels of *PDCD1* and *HAVCR2*, was increased in treatment-naive BMRCC samples (Fig. 4d and Supplementary Fig. 5d). Specifically, CD8-Tex of late-stage primary ccRCC exhibited higher expression of *ENTPD1*, which is related to terminal differentiation of T cells^33^. By contrast, *PDCD1*, *CXCL13*, and *LGALS3* were highly expressed in the CD8-Tex of BMRCC (Supplementary Fig. 5d), indicating the influence of metastatic niche in T cell subsets. In addition, CD8-Tex cells in the BMRCC samples highly expressed genes associated with interferon response, e.g., *CCL5*, *CCL3*, and *ISG15* (Fig. 4f). This observation was consistent with previous findings that intrinsic type I interferon signaling of CD8^+^ T cells skewed the differentiation to a terminal exhaustion state^34^.

Interestingly, we found that the proportions of CD8-Tex were decreased and CD8-Tem was increased, especially CD8-Tem1, in the BMRCC after treatment (Fig. 4d), which was verified by immunostaining assay (Supplementary Fig. 5e). Thus, we next explored the alteration of T cell profiles upon the treatment of PD-1 inhibitor in the BMRCC, and found that CD8-Tex cells in the treated samples showed much higher expression of T cell activation associated genes, including *JUNB*, *CEBPB*, and *HLA-DQB1* (Fig. 4f). Further cell trajectory analysis using Monocle2^35^ revealed that CD8-Tem2 and CD8-Tem1 cells could differentiate into CD8-Tex cells in the treatment-naïve BMRCC (Fig. 4g). While, the CD8-Tex cells in the treated BMRCC patients demonstrated potential to differentiate back into CD8-Tem2 through CD8-Tem1 cluster (Fig. 4h). Moreover, the expression of CD8-Tem1 marker genes were higher in ccRCC patients of stage III/IV (Supplementary Fig. 5f). Survival analysis indicated that high expression level of CD8-Tem1 markers in primary ccRCC was significantly associated with poor prognosis (Supplementary Fig. 5g), whereas the high level of CD8-Tem2 indicated better prognosis (Supplementary Fig. 5h). These results suggested that immunotherapy might reshape the differentiation trajectory of CD8^+^ T cells into activated effector T cells in the bone metastases.

To identify potential transcription factors (TFs) associated with the two different transition directions of CD8-Tem1 cells, we then performed gene regulatory network analysis using SCENIC^36^ and uncovered a series of regulons differentially expressed in CD8-Tem1 cells between the treatment-naïve and treated BMRCC (Fig. 4i). Specifically, *ZFP30*, *ZNF569*, *TCF7*, *MYBL1*, and *HOXB2* were predominantly present in treatment-naïve BMRCC, while *XBP1*, *BCL3*, *ARID5B*, *RELB*, and *NR2C1* were featured by treated BMRCC (Fig. 4i). The targeted genes highly expressed in the CD8-Tem1 of treatment-naïve BMRCC were enriched in cellular metabolic processes and regulation of leukocyte differentiation, while those enriched in the treated group were associated with functions including cytosine signaling in immune system, T cell activation, immune effector process and type I interferon signaling pathway (Fig. 4j). Together, these results indicated that BMRCCs were enriched with infiltrated T cell subtypes with distinct status from the primary ccRCC, and treatment might affect the trajectory path of CD8-Tem cells by activating TFs for T cell activation in the BMRCC.

### Identification of myeloid cell subsets in primary and BMRCC

**Fig. 5:**
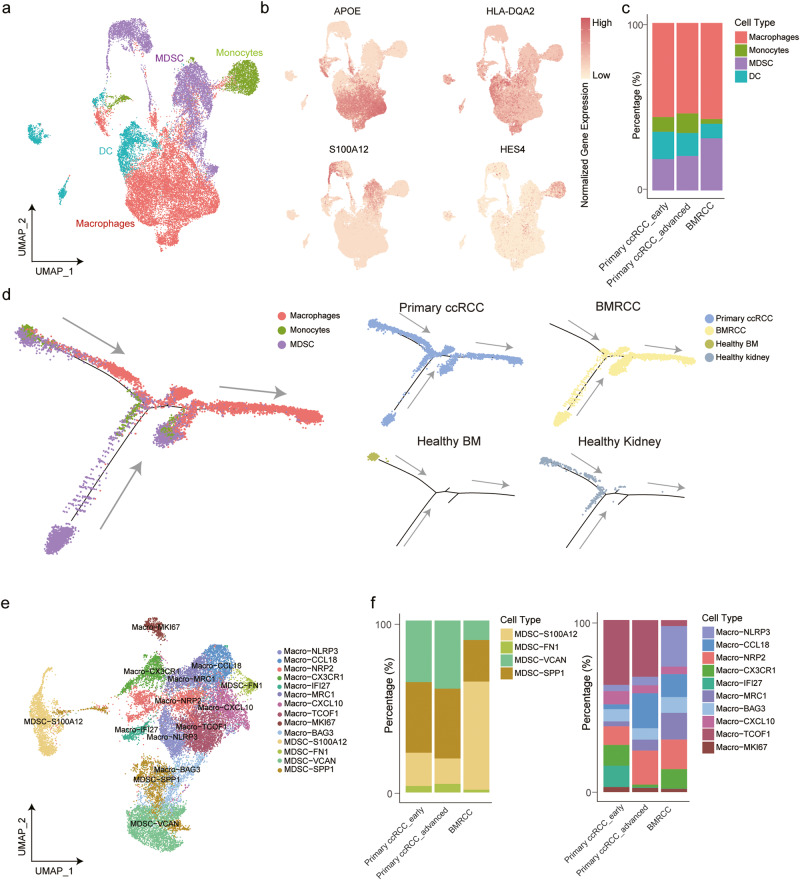
Different trajectory paths of macrophages between the primary ccRCC and BMRCC. **a** UMAP plot showing the four myeloid cell types in the cohort. The colors of the cell indicate the cell type marked by legend. **b** UMAP plot displaying the marker genes of macrophages (*APOE*), DC (*HLA-DQA2*), MDSC (*S100A12*), and monocytes (*HES4*). Orange color indicates higher expression of these genes, pale yellow indicates lower expression of these genes. **c** Bar plot showing the fractions of macrophages, DC, MDSC, and monocytes in the primary ccRCC of early and advanced stages and BMRCC. Colors on the columns indicate cell type marked by legend. **d** Developmental trajectory of macrophages, MDSC, and monocytes in the primary and bone metastatic ccRCC. Colors of cell indicate cell type marked by legend. **e** UMAP plot showing the MDSC and macrophage subtypes by re-clustering analysis. Colors of cell indicate cell type marked by legend. **f** Bar plot showing the fractions of MDSC and macrophage subtypes in the primary ccRCC of early and advanced stages and BMRCC. Colors on the columns indicate cell type marked by legend.

To generate a deeper transcriptional landscape of tumor-infiltrating myeloid cells, which modulate key cancer-associated activities and comprise various subsets with divergent functions, including immune evasion and responses to different types of cancer therapy^37^. We further explored the subpopulations of myeloid cells and identified 4 major lineages (Fig. 5a). Myeloid-derived suppressor cells (MDSC), macrophages, dendritic cells (DC), and monocytes showed high expression of canonical cell markers, including *S100A12*, *APOE*, *HLA-DQA2*, and *HES4*, respectively (Fig. 5b). We next examined the composition of major lineages of tumor-infiltrating myeloid between the primary ccRCCs and BMRCCs (Fig. 5c). Monocytes and DCs were abundant in primary ccRCC, whereas MDSCs and macrophages were increased in BMRCC (Fig. 5c), supporting the notion that MDSCs are generated in the bone marrow from common myeloid progenitor cells^38^.

Macrophages have been reported to differentiate from monocytes in primary tumors^23^, we next explored whether the differentiation trajectory of macrophages in the BMRCC remained the same as the primary ccRCC. Trajectory analysis using the identified MDSCs, monocytes and macrophages in primary ccRCC and BMRCCs as well as normal bone marrow tissues revealed a complete picture of the differentiation trajectories of myeloid cells in the ccRCC (Fig. 5d). The results showed that macrophages in healthy kidney, early- and late-stage primary ccRCC and healthy bone marrows are mostly differentiated from monocytes, while macrophages are mainly derived from MDSC in the BMRCC (Fig. 5d). These results revealed enrichment of different myeloid subtypes together with distinct differentiation trajectories in the BMRCC in comparison with the primary ccRCC.

**Fig. 6:**
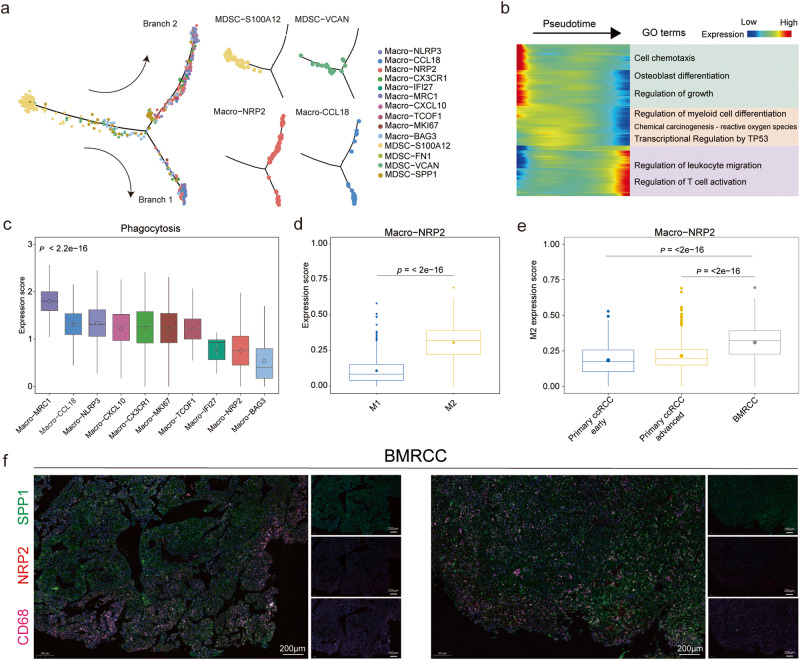
The differential trajectory of myeloid cell subtypes in the BMRCC. **a** Developmental trajectory of MDSC and macrophage subtypes in the BMRCC. Colors of cell indicate cell type marked by legend. **b** The expression patterns of genes associated with the pseudotime and their functional enrichment. Colors on heatmap indicate the genes expression. Color from red to blue indicates a high to low expression. **c.** The expression levels of phagocytosis signatures among the macrophage subtypes in BMRCC. Colors on the columns indicate cell type marked by *x*-axis. *P* values were calculated by Kruskal−Wallis test, *n* = 5172 biologically independent cells. **d** Comparison of the M1- and M2-associated signature gene expression levels in the Macro-*NRP2* cells in BMRCC. Blue columns indicate M1 type, yellow columns indicate M2 type. *P* values were calculated by Student’s *t* test, *n* = 887 biologically independent cells. Effect size of Cohen’s d: 1.826. **e** The expression levels of M2-associated signature genes in Macro-*NRP2* among the primary ccRCC of early and advanced stages and BMRCC. Blue columns indicate primary ccRCC early group, yellow columns indicate primary ccRCC advanced group. The gray columns indicate BMRCC group. *P* values were calculated by Student’s *t* test, *n* = 1833 biologically independent cells. Effect size of Cohen’s d: primary ccRCC early group vs BMRCC: 1.059; primary ccRCC advanced group vs BMRCC: 0.811. **f** mIHC showing the existence of Macro-*NRP2* in BMRCC. The green color indicates the expression of SPP1 protein. The red color indicates the expression of NRP2 protein. The purple color indicates the expression of CD68 protein. Box-and-whisker plots (**c**–**e**): centre line indicates median, box represents first and third quantiles, and whiskers indicate maximum and minimum values.

Further clustering of the bone metastasis-enriched tumor-infiltrating myeloid cells MDSCs and macrophages gave rise to 14 sub-populations with specific gene signatures, including 4 groups of MDSCs and 10 subtypes of macrophages (Fig. 5e, f, Supplementary Fig. 6a–c). We then compared infiltration of subgroups of myeloid cells between primary ccRCC with BMRCC (Supplementary Fig. 6a, b). We observed that MDSC-*S100A12* and Macro-*NLRP3*, Macro-*MRC1*, Macro-*CX3CR1*, Macro-*BAG3*, Macro-*CCL18*, and Macro-*NRP2* were enriched in the BMRCC, with primary ccRCC from early and late stages as comparison (Supplementary Fig. 6a, b). MDSCs were reported to have the ability to markedly influence the trajectory of malignant diseases^38,39^. We then explored the cell transformation relationship between MDSC and macrophage subtypes in the BMRCC, and found that MDSC subgroups MDSC-*S100A12* and MDSC-*VCAN* were the potential origins of macrophages, which were further divided into two branches with different macrophage subgroups (Fig. 6a and Supplementary Fig. 6d). Notably, genes highly expressed at the start of the trajectory were enriched in GO terms including chemotaxis, regulation of growth and osteoblast differentiation (Fig. 6b), consistent with the upregulated expression of *S100A9*, *S100A12*, *S100A4* and *S100A6* in the MDSC-*S100A12* and MDSC-*VCAN* subtypes (Supplementary Fig. 6e). The expression of genes related to regulation of myeloid cell differentiation were increased at the transformation stage, accompanied by the transcriptional regulation by *TP53* (Fig. 6b and Supplementary Fig. 6e). Genes including *APOE*, *CD81*, *CD9* and *GPNMB* showing high expression levels at the end of differentiation process were significantly enriched in leukocyte migration and T cell activation pathways (Fig. 6b and Supplementary Fig. 6e), suggesting potential interaction between macrophages and T cells in the BMRCC.

By defining the dichotomous M1/M2 dualistic polarization state and functional phenotypes of macrophages subtypes, we found that Macro-*CCL18* and Macro-*MRC1* subgroups, which differentiated in the same branch (Branch 1) at the developmental trajectory, exhibited higher M2 signature and preferential expression of genes involved in phagocytosis (Fig. 6a, c). For the subtypes of macrophages on Branch 2, Macro-*NLRP3* showed higher expression of phagocytosis signatures (Fig. 6a, c), whereas Macro-*NRP2* exhibited higher pro-angiogenic signatures and M2 signature (Fig. 6a, d and Supplementary Fig. 6f). Besides, the M2 signature expression levels of Macro-*NRP2* in the BMRCC were significantly higher than primary ccRCC of both early and late stages (Fig. 6e). In addition, high expression of Macro-*NRP2* was associated with poor prognosis of primary ccRCC patients (Supplementary Fig. 6g). The existence of Macro-*NRP2* in BMRCC was showed by mIHC (Fig. 6f). These results indicated that Macro-NRP2 had anti-inflammatory and M2 polarization characteristics in the BMRCC, and could also be used as a predictive marker for prognosis of ccRCC.

### Interaction between macrophages and T cells in the bone metastatic environment

**Fig. 7:**
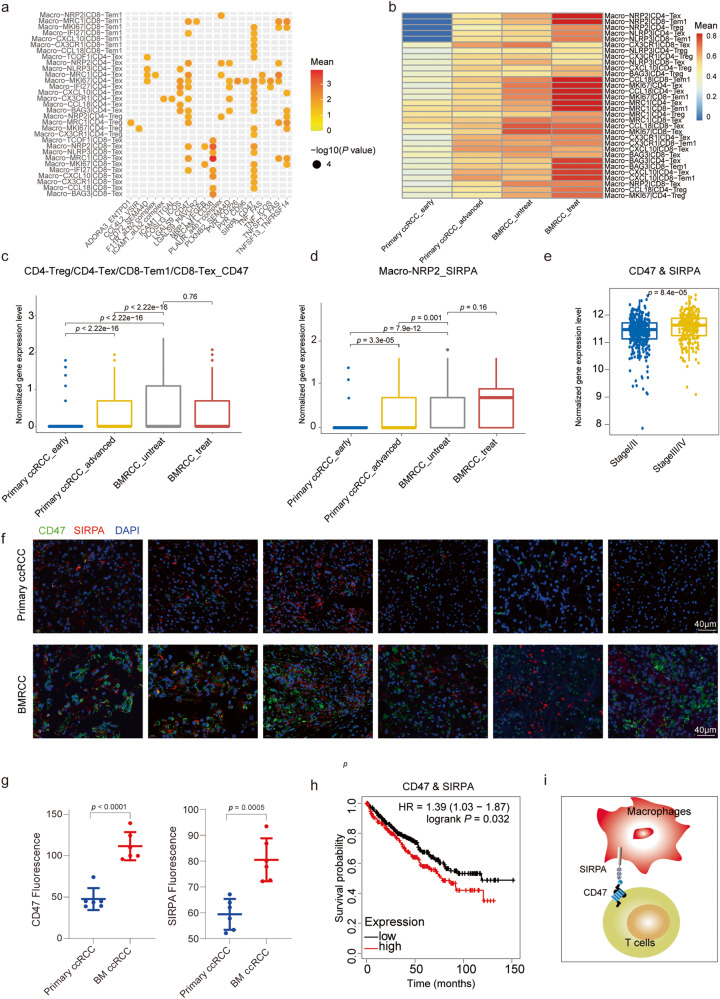
Cell-cell communication between macrophages and immune inhibitory T cells. **a** Bubble plot showing the interaction activities of different ligand-receptor pairs across macrophage subtypes and immune inhibitory T cell subtypes. Dot size indicates –log 10 (*P* value). Dot color indicates mean of interaction activities. Dot color from red to yellow indicates a high to low interaction activity. **b** Heatmap displaying the interaction activities of *SIRPA*-*CD47* pair between macrophage and immune inhibitory T cell subtypes. Colors on heatmap indicate mean of *SIRPA*-*CD47* interaction activities. Color from red to blue indicates a high to low interaction activity. **c** Comparison of *CD47* expression levels in the inhibitory T cell subtypes among primary ccRCC of early and advanced stages and BMRCC with or without treatment. Blue columns indicate primary ccRCC_early group, yellow columns indicate primary ccRCC_advanced group, gray columns indicate BMRCC_Untreat group, red columns indicate BMRCC_Treat group. *P* values were calculated by wilcox rank sum test, *n* = 24157 biologically independent cells. Effect size of Cohen’s d: BMRCC_Treat vs BMRCC_Untreat: 0.027; primary ccRCC early vs BMRCC_Untreat: 0.516; primary ccRCC_advanced vs BMRCC_Untreat: 0.292; primary ccRCC_early vs primary ccRCC_advanced: 0.282. **d** Comparison of *SIRPA* expression levels in the Macro-*NRP2* subtype among primary ccRCC of early and advanced stages and BMRCC with or without treatment. Blue columns indicate primary ccRCC_early group, yellow columns indicate primary ccRCC_advanced group, gray columns indicate BMRCC_Untreat group, red columns indicate BMRCC_Treat group. *P* values were calculated by wilcox rank sum test, *n* = 1833 biologically independent cells. Effect size of Cohen’s d: BMRCC_Treat vs BMRCC_Untreat: 0.666; primary ccRCC early vs BMRCC_Untreat: 0.424; primary ccRCC_advanced vs BMRCC_Untreat: 0.187; primary ccRCC_early vs primary ccRCC_advanced: 0.262. **e** Comparison of *CD47* and *SIRPA* co-expression levels between the early (Stage I/II) and late (Stage III/IV) staged primary ccRCC in TCGA. Blue columns indicate stage I/II samples, yellow columns indicate stage III/IV samples. *P* values were calculated by wilcox rank sum test, *n* = 606 biologically independent samples. Effect size of Cohen’s d: 0.307. **f** Immunofluorescence staining showing the expression of *CD47* and *SIRPA* in the primary ccRCC and BMRCC. The green color indicates the expression of CD47 protein. The red color indicates the expression of SIRPA protein. The blue color indicates nucleus of cell. **g** Barplot showing the fluorescence intensity of CD47^+^ cells and SIRPA^+^ cells in primary ccRCC and BMRCC. Blue dots indicate primary ccRCC samples, red dots indicate BMRCC samples. *P* values were calculated by Student’s *t* test. *n* = 12 biologically independent samples. Effect size of Cohen’s d: CD47: 4.182; SIRPA: 2.912. **h** Kaplan–Meier plots showing the survival probability of primary ccRCC patients with high and low co-expression levels of *CD47* and *SIRPA*. Red line indicates patients expressed higher *CD47* and *SIRPA* co-expression. grey line indicates patients expressed lower *CD47* and *SIRPA* co-expression. *P* values were calculated by log-rank test. **i** The model of the action of macrophages and T cells through *SIRPA*-*CD47*. HR, hazard ratio. Box-and-whisker plots (**c**–**e**): Centre line indicates median, box represents first and third quantiles, and whiskers indicate maximum and minimum values.

Given the dominance of macrophages in the BMRCC and their potential role for regulating T cell activities in the tumor microenvironment^40^, we next explored the cell-cell interactions between macrophage subtypes and immune-inhibitory T cells using CellPhoneDB^41^. Compared with primary ccRCC, we found specific receptor-ligand pairs between macrophages and immune-inhibitory T cells were enriched in BMRCC, including pro-migratory interaction (*CCL4L2*-*VSIR*), and immune-inhibitory interactions (*SIRPA*-*CD47*, *LGALS9*-*HAVCR2*, *LGALS9*-*CD47*, *TNF*-*FAS* and *TNF*-*ICOS*) (Fig. 7a). Among them, the interaction between *SIRPA*-*CD47* pairs was widespread across diverse types of macrophage and inhibitory T cells (Fig. 7a). In addition, the interaction between macrophages and inhibitory T cells though *SIRPA*-*CD47* increased with the malignant progression of ccRCC, with the lowest in the early stage of ccRCC and highest in the BMRCC (Fig. 7b). Further analysis showed that expression levels of *CD47* in immune inhibitory T cells were much higher in BMRCC than those of primary ccRCC, and PD-1 inhibitor treatment could not decrease the *CD47* expression (Fig. 7c and Supplementary Fig. 7a). *SIRPA* was found to be highly expressed in macrophages of BMRCC (Fig. 7d and Supplementary Fig. 7b), and expression level was further elevated in macrophages of treated BMRCC (Fig. 7d and Supplementary Fig. 7b). These results were confirmed by the higher expression of *SIRPA* and *CD47* in patients with late-stage ccRCC (Fig. 7e, Supplementary Fig. 7c, d). Consistently, compared with the primary ccRCC, we observed the simultaneous enrichment of *SIRPA* and *CD47* in the BMRCC by immunofluorescence staining (Fig. 7f, g). Interestingly, the co-expression of *SIRPA* and *CD47* was significantly associated with poor prognosis for ccRCC patients (HR = 1.39, *P* = 0.032) (Fig. 7h), while high expression of *CD47* or *SIRPA* only slightly contribute to poor prognosis of ccRCC patients (HR = 1.34, *P* = 0.054 for *CD47*; HR = 1.33, *P* = 0.078 for *SIRPA*) (Supplementary Fig. 7e, f). Taken together, the expression of *SIRPA*-*CD47* pair could serve as a potential bone metastasis signal with poor prognosis for RCC (Fig. 7i).

## Discussion

Here, we provide a high-resolution landscape of human primary (early and advanced stages) and BMRCC, and the underlying molecular mechanisms for bone metastasis of RCC. Based on the scRNA-seq data, we compared the transcriptomic profiles of cancer cells from primary ccRCC with BMRCCs. We also observed major lineages of cell types together with subtypes of fibroblasts, myeloid cells and T cells that enriched in the BMRCC, which were supported by their association with poor prognosis. The *SIRPA*-*CD47* interaction between myeloid subgroups and specific T cell clusters pointed to molecular-targeted immunotherapy as potential therapeutic solutions to be further explored for the treatment of BMRCC.

As the most common subtype of RCC, previous studies have applied scRNA-seq to identify cell of origin and transcriptomic differences among different cell types in ccRCC^4,42^. Ke et al. reported that hypoxia response, lipid biosynthesis, and localization pathways were enriched in malignant renal cells compared with normal renal cells^43^. Here, we found cancer cells in BMRCC showed stronger migration ability and angiogenesis ability in comparison with primary tumor cells of RCC. Together, the alteration from metabolic capacity to migratory capacity might point to the malignant evolution of renal tumor cells to RCC metastasis.

Our scRNA-seq analysis revealed that genes enriched in tumor cells with evident copy number amplifications were associated with the *WNT* signaling pathway in the bone metastatic environment. *WNT* signaling is critically involved in both the development and homeostasis of tissues via regulation of their endogenous stem cells^44^. In tumor microenvironment, aberrant *WNT* signaling was considered to play a key role in the initiation, maintenance and development of multiple cancers by regulating the behavior of cancer stem cells^45,46^. Previous studies have demonstrated *WNT* signaling pathway as a good therapeutic target for primary renal cancer^47,48^. Potential therapeutic reagents targeting this pathway might work as efficient treatment solutions for BMRCC in clinic.

CAFs are the most prominent stromal components in solid tumors and rarely studied in RCC. Four transcriptionally diverse subpopulations of CAFs were defined in breast cancer^49^, which three groups were also found in primary ccRCC and BMRCCs (Fig. 3). The iCAF cluster was a specific CAF subpopulation found in primary ccRCC and BMRCCs, and it was also described in pancreatic ductal adenocarcinoma^30^. Interestingly, we found a mCAF cluster (mCAF_2) with high expression of *FAP* and *MMP13* dominant in BMRCC (Supplementary Fig. 4g). *FAP* is a dimeric Type II transmembrane glycoprotein with proteolytic activity, and was reported to be highly expressed in tumor stroma^50^. *FAP* was also considered as a promising target for radionuclide-based approaches for diagnosis and treatment of tumors^51^. Our results here showed that *FAP*^+^ CAFs might be associated with bone metastasis of RCC, and the underlying mechanism deserves further investigation.

T cells are the most abundant and best-characterized population in the tumor microenvironment of solid tumors. Here we revealed the dominance of inhibitory T cells and higher expression of exhaustion-related genes in the BMRCC, consistent with previous findings that exhausted T cells are enriched in advanced tumors^52,53^. In addition to the *PDCD1*, the *LAG3* and *CXCL13* were also overexpressed in the bone metastatic samples. Previous studies have shown that *CXCL13* could reshape the lymphoid structures and promote response to immunotherapy in multiple advanced cancers^54–56^. Thus, restoring the exhausted T cells provides a promising strategy for preventing tumor progression. Our results showed that the trajectory path of CD8-Tem1 reversed from CD8-Tex and differentiated into CD8-Tem2 cells after treatment, suggesting the effectiveness of PD-1 inhibitor to the BMRCC. The distinct differentiation directions of CD8-Tem1 in the BMRCC after treatment might be partially explained by the differential activation of TFs and their downstream gene expressions, which needs to be further explored for better understanding of potential regulatory mechanisms.

In addition, we also found the enrichment of MDSCs and macrophages in the bone metastatic tumors of RCC. The MDSCs are a population of myeloid cells and immature myeloid cells could convert to immunosuppressive MDSCs under pathologic conditions^57^. The abundance of MDSCs in the BMRCC might represent a pathogenic state of activation of monocytes in the bone metastasis environment. Our trajectory analysis revealed that both MDSCs and monocytes are both progenitors of macrophages, and macrophages differentiate from monocytes in the primary ccRCC, consistent with previous reports^24,58]^. The differentiation trajectory of macrophages from MDSCs in the BMRCC was also different from that of normal bone marrows, hinting that targeting MDSC may provide tangible clinical benefits in the BMRCC. We also identified a bone metastasis-enriched macrophage subtype with high expression of *NRP2* gene, which is a single transmembrane receptor and plays a key role in promoting tumor proliferation, invasion and metastasis by interacting with vascular endothelial growth factors^58,59^. *NRP2* is not detectable in the bone marrow or monocytes of humans^60,61^, and the expression of *NRP2* in myeloid cells is upregulated during the differentiation to macrophages^62^. Consistent with our findings that Macro-*NRP2* was characterized by high expression of M2 macrophage features, a previous study reported that reduced expression of *NRP2* after LPS stimulation of macrophages triggers M1 polarization^63^. Given the M2 polarization and pro-angiogenesis features of Macro-*NRP2* and its potential in regulating immune inhibitory T cells in the BMRCC, this macrophage subgroup emerged as a potential therapeutic target for further investigations in the BMRCCs. The *CD47* expression on inhibitory T cells might inhibit macrophage-mediated elimination in a manner that bears a superficial resemblance to the inhibition of macrophages by *CD47*^+^ cancer cells^15,64^. The inhibition of macrophage function favors the survival of inhibitory T cells and cancer cells, which in turn contributes to the malignancy of tumors. Thus, the interaction between macrophages and inhibitory T cell clusters through *SIRPA* ligand and *CD47* receptor serves as an alternative way for understanding the bone metastasis of RCC.

Limitations of this study include unpaired primary and bone metastatic biopsy tissues and the small sample size. The uncertainty in the development of bone metastatic tumors from the initial treatment of ccRCC limited the ability to collect paired primary and BMRCC samples from the same patient in clinical practice. The findings might thus be confounded by the variations of genetic background and somatic mutations. Although the inclusion of single-cell data from late-stage primary ccRCC, normal kidney and healthy bone marrow tissues could reduce some of the confounding factors, further studies are needed to investigate the gene signatures in patients after controlling such clinical variables. Besides, the small sample size of the cohort limited the power of statistical significance in the results. Biological variation between samples may be confusing some of the results and more study samples will be needed in the future to confirm these results. In addition, the BMRCC patients included in our cohort are treated with immunotherapy and TKIs, which hinders the possibility to explore the influence of single regimen. The treatment of collected samples are anti-vascular and anti-PD-1 therapy, so we mainly included the treatment for stratification in these cell cancer cells and T cells. The effect of these treatments on other cell types like CAF and macrophages might be indirect and complicated, further studies are needed for better understanding of different treatment options on the microenvironment of BMRCC. Furthermore, despite of the survival analysis using TCGA cohort suggested that the signatures enriched in BMRCC might be markers indicating malignant tumor progression, our results have shown BMRCC was different from late-stage primary ccRCC (Supplementary Fig. 2d, 5d, Fig. 5c, f, and Fig. 6e), raising the necessity of generating large cohorts of BMRCC samples for further exploration.

In summary, our comprehensive characterization of cell landscape of BMRCC revealed that intra-tumoral heterogeneity of primary and bone metastatic ccRCC. Our study identified key cell subsets and molecular features enriched in the bone metastatic environment of ccRCC. The development trajectory and cell-cell interaction analyses also revealed immune cell subtypes served as targets for BMRCC. Although the descriptive nature of this study, our data offer a rich resource to better understand various cell types in BMRCCs and thus provide valuable insights for therapeutic solutions.

## Methods

### Patient information

Fourteen patients who were pathologically diagnosed with clear cell renal cell carcinoma, were enrolled in this study. A total of fifteen samples were obtained with two bone metastatic samples (P11_M1, P11_M2) were collected from one single patient. Six out of fifteen samples were collected from primary tumor site and nine of fifteen samples were collected from bone metastatic tumor site. Their clinical characteristics are summarized in Supplementary Table 1. All samples were discarded tissue after surgery. Written informed consent was provided from each patient prior to sample collection. This study was reviewed and approved by the Institutional Review Board of Fudan University Shanghai Cancer Center.

### Sample collection

The femur metastasis sample was resected from tumor mass around the bone after pathological fracture, and only metastasis tumor sample was obtained to avoid potential influence of bone healing process. Similarly, surgical resection of rib metastasis included resection of the affected rib, adjacent muscles, and any other tissues adherent to the tumor. The specimen was also obtained from tumor mass without involvement of the osseous tissue. For the sacral and spinal (vertebral) sample, en bloc resection was performed in those patients to minimize the tumor residue, following a posterior spinal fixation using spinal instruments. In most patients, the pedicle screw fixation was used for posterior stabilization, in order to achieve biomechanical stability after vertebral resection.

### Single-cell isolation, cDNA amplification and library construction

Fresh samples isolated from patients in operation were preserved in MACS Tissue Storage Solution (Miltenyi Biotec) at 4 °C. Tumor tissues were cut into a range of 0.2–1.0 g small pieces and dissociated in 5 mL enzyme mix containing 4.7 ml RPMI 1640 (Gibco), 200 μL Enzyme H, 100 μL Enzyme R and 25 μL Enzyme A (Miltenyi Biotec, MACS Tumor Dissociation Kit, human). The samples were subsequently incubated in a 37 °C thermostatic shaker for 35 min. Then suspended samples were filtered through a 40-μm Cell-Strainer nylon mesh (BD) with 30 mL of RPMI 1640 and centrifuged at 300 × g for 7 min. After removing the supernatant, we used Red Blood Cell Lysis Solution (Miltenyi Biotec #130-094-183) and the Dead Cell Removal Kit (Miltenyi Biotec #130-090-101) to remove red blood cells and obtain live cells. Cell suspension was centrifugated at 300 × g for 7 min and the pellet was re-suspended in 1 mL PBS solution. Once the desired cell suspension was obtained, the sample was immediately placed on ice for subsequent GEMs preparation and reverse transcription. The single cell libraries were prepared according to the standard protocols and sequenced on Illumina NovaSeq 6000 Systems using paired-end sequencing (150 bp in length).

### Single-cell RNA-seq data processing

The scRNA-seq data generated from the 10× Genomics platform were aligned and quantified using CellRanger (version 6.0.2) against the GRCh38 human reference genome. A raw gene expression matrix for each scRNA-seq sample was generated by CellRanger. Cell-free RNA was removed using SoupX (version 1.5.2), and doublets were predicted and filtered using DoubletFinder (version 2.0.3). Then these matrices were combined as an integral gene expression matrix for all samples using the Seurat package (version 4.0.5) implemented in R (version 4.1.0). Further quality control was applied to cells, cells with less than 500 detected genes, more than 8000 detected genes, 20000 UMI counts and 10% mitochondrial gene count were filtered (Supplementary Fig. 2a). RunHarmony function in R Seurat package was applied to remove batch effects between data from different sources. The integrated gene expression matrix was used for the downstream analyses. The differences in cell abundances among samples and groups was calculated using the Milo framework^65^.

### Identification of the major cell types and their subtypes

The Seurat R package was applied to identify major cell types. First, scTransform function^66^ was used to normalize the influence of sequencing depth, mitochondria and other factors. Then 3000 highly variable genes were generated, and used to perform principal component analysis (PCA). The top 30 principal components were calculated to reveal the main axes of variation and denoise the data. Cells were clustered by unsupervised graph-based clustering algorithm using their expression profiles. For visualization, UMAP and t-SNE dimensionality reduction were applied by using RunUMAP and RunTSNE functions. The cluster-specific marker genes were identified by running FindAllMakers function with default parameters. Ten major cell types were identified: T cells (*CD3D*, *CD3E*), nature killer T-like (NKT) cells (*GNLY*, *FGFBP2*), myeloid cells (*CD14*, *FCGR3A*, *LYZ*), nature killer (NK) cells (*XCL2*, *KLRC1*), endothelial cells (*PLVAP*, *PECAM1*), cancer cells (*KRT18*, *VEGFA*), cancer-associated fibroblasts (CAFs) (*COL1A1*, *COL1A2*), B cells (*CD79A*, *MS4A1*), mast cells (*TPSB1*, *TPSAB1*), and neutrophils (*S100A8*, *S100A9*). Second, to identify subclusters within major cell type, the cells belonging to each cell type were re-analyzed separately with scTransform, dimensionality reduction, and clustering by unsupervised graph-based clustering algorithm. Then the subclusters were annotated to cell subtypes by subcluster-specific marker genes shown in the corresponding figures and Supplementary Data 1.

### Tissue distribution of clusters

The ratio of observed to expected cell numbers (Ro/e) was calculated for each cell type or subtype between primary ccRCC and BMRCC to quantify the tissue preference of each cell type or subtype^67,68^. The expected cell numbers for each combination of cell type or subtype and tissues were obtained from the chi-square test. Ro/e > 1 suggested that one cell type or subtype was identified as being enriched in a specific tissue.

### Copy number variations analysis for tumor cells

To further investigate genetic heterogeneity between tumor cells in primary and bone metastatic tumors, inferCNV (https://github.com/broadinstitute/inferCNV) was used to infer copy-number alteration for all the tumor cells. The copy number variations scores of the fibroblasts and endothelial cells were also calculated as a copy number variations control. Then the whole copy number variations profiles were normalized by subtracting the average expression profiles of control. The scores were restricted to the range −1 to 1 by replacing all values >1 with 1 and all values <−1 with −1, and any score between −0.3 and 0.3 was set to 0.

### Transcription factor analysis

Activated TFs regulons in each CD8^+^Tem subset were analyzed using SCENIC^36^. The pySCENIC package (version 0.11.2) was applied with raw count matrix as input. Briefly, the regulons were identified by RcisTarget and the co-expression network was calculated using GRNBoost2. Next, the regulon activity foreach cell was scored by AUCell.

### Trajectory analysis

To characterize the developmental state of MDSC and macrophages, the Monocle (version 2.20.0) algorithm^35^ was applied with significant genes (*q* < 0.05, top 3000 genes) of the studied cells were identified by using the differentialGeneTest function. Cell differentiation trajectory was constructed on these signature genes with the default parameters of Monocle after dimension reduction and cell ordering.

### Polarization state and functional phenotypes analysis of macrophages subtypes

To further define dichotomous M1/M2 dualistic polarization state and functional phenotypes of macrophages subtypes, gene sets associated with M1/M2 state and angiogenesis/Phagocytosis phenotypes (Supplementary Table 3) were analyzed by comparing the mean expression values of cells in each macrophages subtype.

### Cell–cell communication analysis

To explore the potential interactome between different cell types in RCC tumor micro-environment, the CellPhoneDB algorithm^41^ was used to infer cell-cell communication. Single-cell transcriptomic data of all macrophage subtype and immune inhibitory T cells (CD4-Treg, CD8-Tex, CD8-Tem1) was analyzed by using CellPhoneDB package (version 3.0.0). The mean value of interactions was assessed for BMRCC vs primary ccRCC.

### Function analysis

Metascape^69^ (https://metascape.org/gp/index.html) was used for functional enrichment of different gene sets. The GSVA R package (version 1.40.1)^70^ from Bioconductor was used to assign pathway activity (c2BroadSets), which were described in the molecular signature database^71^. Gene Set Enrichment Analysis (GSEA) in the clusterProfiler R package (version 4.0.5)^72^ to evaluate the activation of hallmark pathways from the molecular signature database.

### Bulk RNAseq datasts analysis

RCC expression data, mutation information and clinal information were performed using TCGAbiolinks R packages^73^. For survival analysis, the top ten significant genes of each cell subtype were used as gene set to evaluate the correlation between each cell subtype and the survival state of RCC patients by Kaplan-Meier Plotter (https://kmplot.com/analysis/index.php?p=background). Mutation analysis was performed using maftools R package (version 2.8.5)^74^. The RCC FPKM data was analyzed to compare the expression levels of genes (*CD47*, *SIRPA*, and the mean of *CD47* and *SIRPA*) at different clinical stages for RCC patient.

### Immunofluorescence staining

Formalin-fixed, paraffin-embedded (FFPE) tissues containing primary and bone metastatic tumors of RCC were sliced into 4 μm sections and stained with antibodies against FAP (abcam, ab218164, Rabbit pAb, 1:1000), Vimentin (CST, 5741, Rabbit mAb, 1:1000), CD8A (abclonal, A0663, Rabbit mAb, 1:1000), PD-1 (abclonal, A20217, Mouse mAb, 1:1000), GZMB (abclonal, A22993, Rabbit mAb, 1:1000), CD68 (abclonal, A23205, Rabbit mAb, 1:1000), NRP2 (proteintech, 11268-1-AP, Rabbit pAb, 1:1000), SPP1 (proteintech, 22952-1-AP, Rabbit pAb, 1:1000), CD47 (SCBT, sc-12730, Mouse mAb, 1:500), and SIRPA (abcam, ab260039, Rabbit mAb, 1:1000) according to the standard protocols.

### Statistics and Reproducibility

Mann-Whitney U test and Student’s *t* test for non-parametric samples were used to calculate *p* values between the two groups. For TCGA datasets, *P* values between two conditions were adjusted for multiple test corrections using the Benjamini–Hochberg algorithm to control the false discovery rate using DESeq2.

### Reporting summary

Further information on research design is available in the Nature Portfolio Reporting Summary linked to this article.

### Supplementary information

Supplementary Information

Description of Additional Supplementary Files

Supplementary Data 1

Supplementary Data 2

Reporting Summary

## Data Availability

The scRNA-seq dataset of primary and bone metastasis RCCs developed by this study are available at the National Omics Data Encyclopedia (NODE) under accession number OEP004678^75^ (https://www.biosino.org/node/project/detail/OEP004678). Other sequencing data that support the findings of this study have been deposited in the National Center for Biotechnology Information Gene Expression Omnibus (GEO)^76^ under the GEO Series accession number: GSE120221, GSE131685, the website under address: https://singlecell.broadinstitute.org/single_cell/study/SCP1288/tumor-and-immune-reprogramming-during-immunotherapy-in-advanced-renal-cell-carcinoma#study-summary, and paper supplementary files under address: https://www.sciencedirect.com/science/article/pii/S153561082100115X?via%3Dihub#sec5.2. Source data are included in Supplementary Data 2.

## Code availability

All computer code used in this study is publicly available. The code can be obtained by visiting 10.5281/zenodo.10321536^77^.
