# Supplementary material for: Single-cell profiling of the microenvironment in human bone metastatic renal cell carcinoma
Source: Commun Biol. 2024 Jan 12;7:91. doi: 10.1038/s42003-024-05772-y (PMC10786927; doi:10.1038/s42003-024-05772-y)
Supplement: Supplementary file 1 — Supplementary Information [file 42003_2024_5772_MOESM1_ESM.pdf]

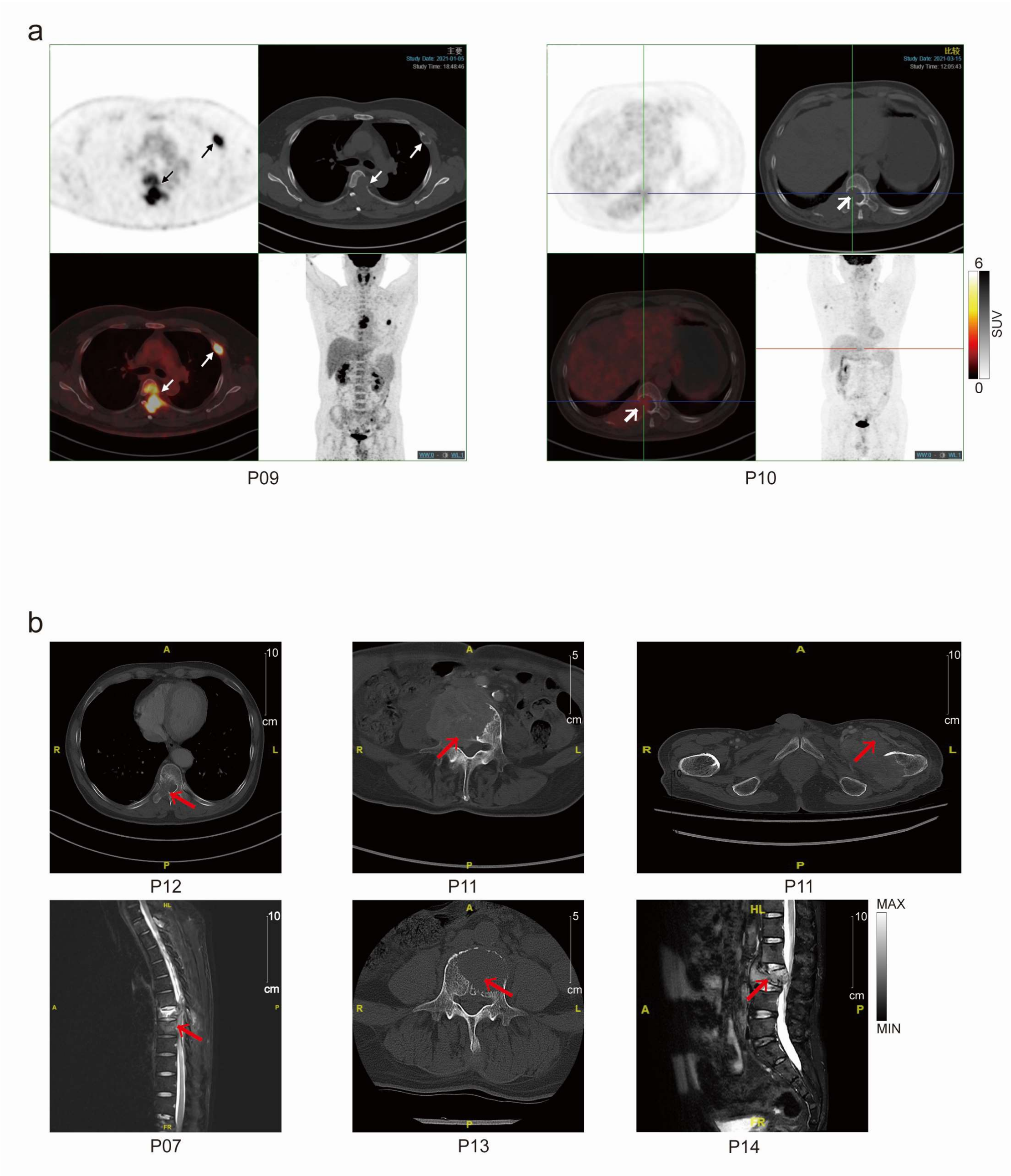

Supplementary Figure 1. Representative PET-CT, MRI and CT results of BMRCC patients.

a. The positron emission tomography–computed tomography (PET-CT) results showing the exact positions of the bone metastatic tumors for two representative BMRCC patients. The white arrows indicate the bone metastases. b. The magnetic resonance imaging (MRI) and computed tomography (CT) results for the bone metastatic tumors of five BMRCC patients in this study. The red arrows indicate the bone metastatic tumor tissues. SUV: standard uptake value.

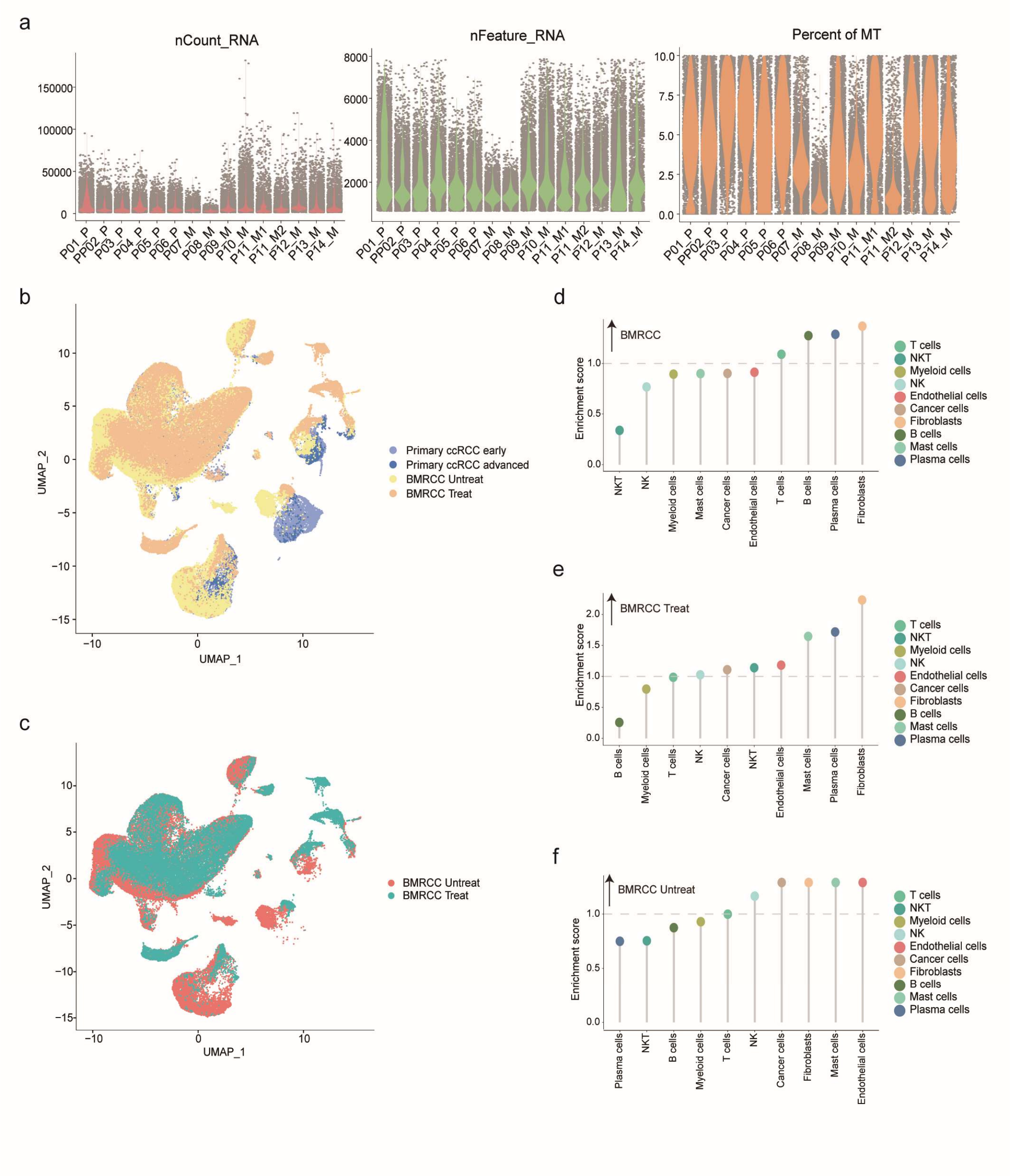

Supplementary Figure 2. The overview of scRNA-seq data used in this study.

a. Violin plots showing the number of counts, the number of genes and the ratio of mitochondrial genes in each cell of each sample after quality control. b. UMAP plot displaying the cell origins from primary ccRCCs of early and advanced stages and BMRCC with or without treatment. Colors of the cell indicate the cell type marked by legend. c. UMAP plot showing single cells of BMRCC patients with or without treatment. Colors of the cell indicate the cell type marked by legend. d. Lollipop plot showing the tissue distribution of each major cell type by Ro/e analysis in advanced primary ccRCCs and BMRCCs. Dot colors indicate the cell type labelled. BMRCC-enriched cell types were characterized with Ro/e > 1. e. Lollipop plot showing the tissue distribution of each major cell type by Ro/e analysis in BMRCCs with and without treatment. Dot colors indicate the cell type labelled. Cell types enriched in the

BMRCC with treatment were characterized with Ro/e > 1. f. Lollipop plot showing the tissue distribution of each major cell type by Ro/e analysis between the treatment-naïve BMRCCs and healthy bone marrow tissues. Dot color indicate the cell type labelled. Treatment-naïve BMRCC enriched cell types were characterized with Ro/e > 1. Fisher's exact test was used to compare significance.

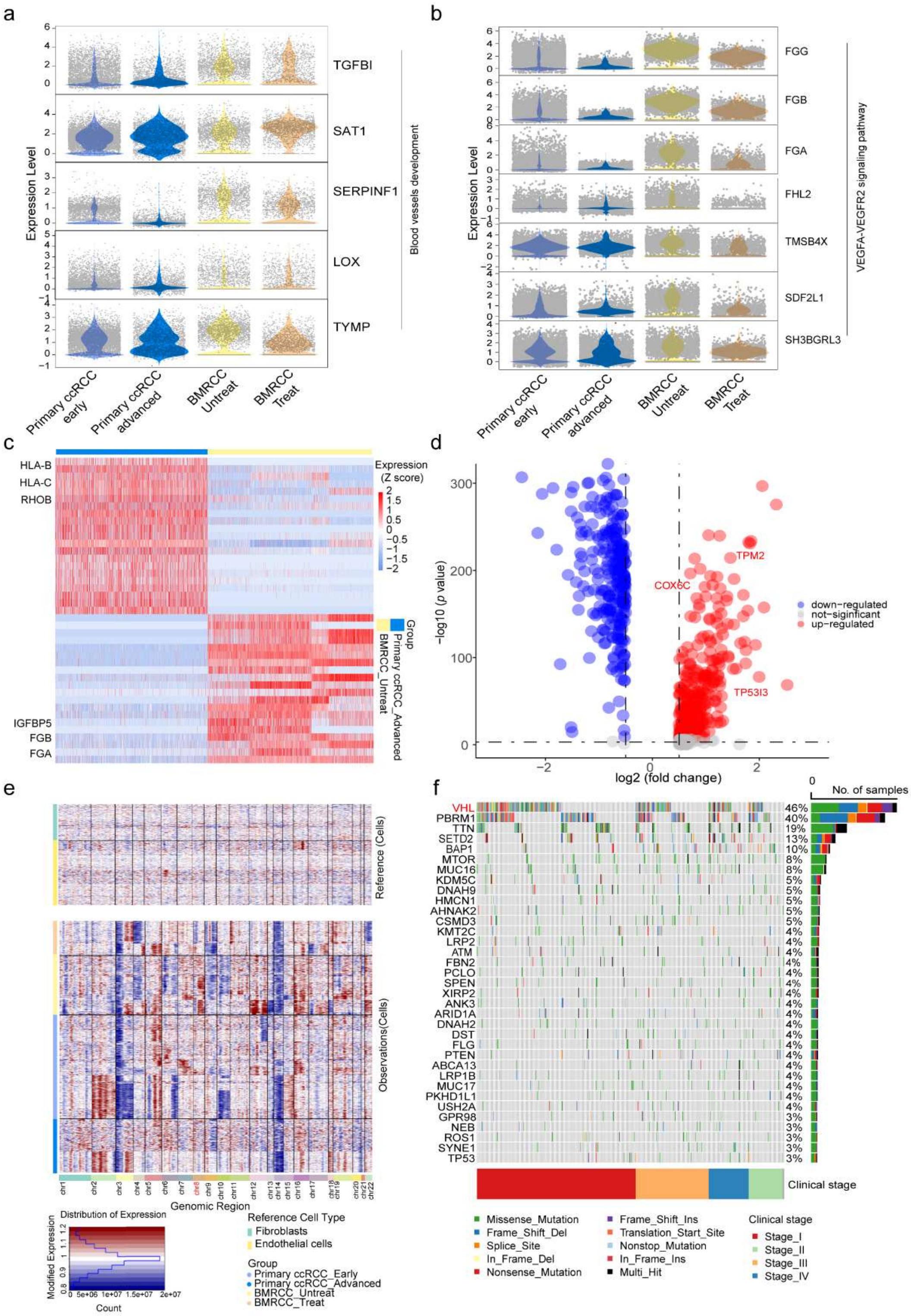

Supplementary Figure 3. Comparison of cancer cell characteristics between primary ccRCCs and BMRCCs.

a. Violin plots showing the expression levels of genes associated with blood vessel development among the indicated ccRCC groups. Colors on the columns indicate groups marked by x-axis. b. Violin plots showing the expression levels of genes associated with VEGFA-VEGFR2 signaling pathway among the indicated ccRCC

groups. Colors on the columns indicate groups marked by x-axis. c. Heatmap showing the differentially expressed genes between the advanced primary ccRCCs and BMRCCs. The single cells are ordered by their tissue origins marked by the upper legend column. Red color on heatmap indicates higher expression, blue color indicates low expression. d. Volcano plot showing the differentially expressed genes between BMRCCs with and without treatment. Red dots indicate genes highly expressed in BMRCCs without treatment group. Blue dots indicate genes highly expressed in BMRCCs with treatment group. e. Heatmap showing the CNV score per cell across each cancer cell subset estimated by inferCNV. The single cells are ordered by their tissue origins marked by the legend column on the left. Red color on heatmap indicate high CNV score, blue color indicate low CNV score. f. Heatmap displaying the top 20 frequently mutated genes in the TCGA-KIRC data. The single samples are ordered by their clinical stage marked by the legend column below. The colors on heatmap indicate mutation type marked by legend below.

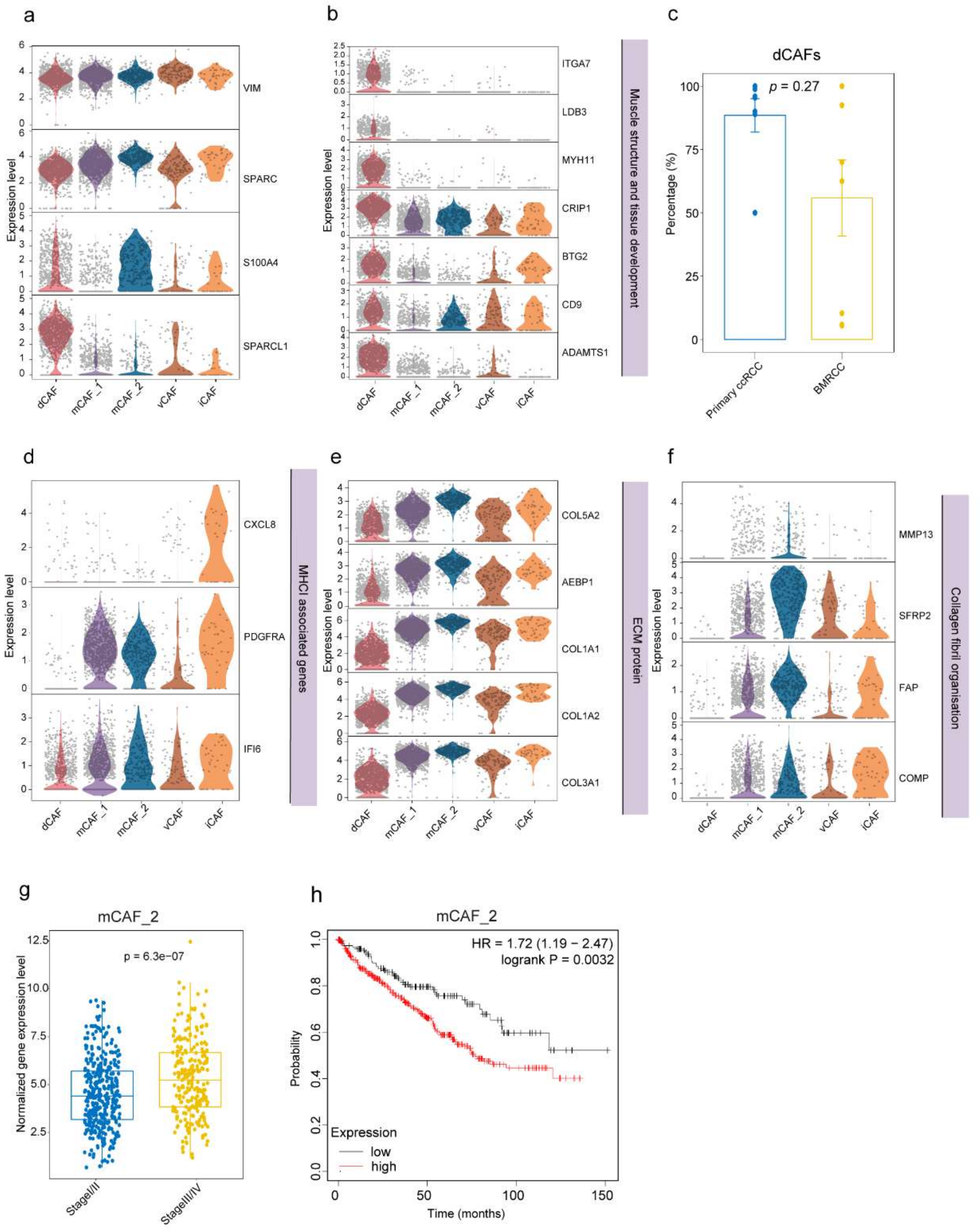

Supplementary Figure 4. Gene signatures of CAF subsets in the primary ccRCC and BMRCC.

a. Violin plot showing the expression levels of common fibroblast marker genes (*S100A4*, *SPARCL1*, *VIM* and *SPARC*) across CAF subsets. Colors on the columns indicate celltypes marked by x-axis. b. Violin plot showing the expression levels of dCAF marker genes across CAF subsets. Colors on the columns indicate celltypes marked by x-axis. c. Violin plot showing the expression levels of iCAF marker genes across CAF subsets. Colors on the columns indicate celltypes marked by x-axis. d. Comparison of the dCAF fractions among primary ccRCC of early and advanced stages and BMRCC. The blue columns indicate primary ccRCC_early group. The yellow columns indicate primary ccRCC_advanced group. The grey columns indicate

BMRCC group. Data presents the mean ± SEM. *P* values were calculated by wilcox rank sum test, n=15 biologically independent samples. Effect size of Cohen's d: 0.979. e. Violin plot showing the expression levels of genes associated with ECM protein among the CAF subclusters. Colors on the columns indicate celltypes marked by x-axis. f. Violin plot showing the expression levels of genes associated with collagen fibril organization among the CAF subclusters. Colors on the columns indicate celltypes marked by x-axis. g. Comparison of mCAF_2 marker genes expression between the early (Stage I/II) and late (Stage III/IV) staged primary ccRCCs using the TCGA-KIRC RNA-seq data. The blue columns indicate stage I/II samples. The yellow columns indicate stage III/IV samples. Centre line indicates median, box represents first and third quantiles, and whiskers indicate maximum and minimum values. *P* values were calculated by Student's t test, n= 606 biologically independent samples. Effect size of Cohen's d: 0.428. h. Kaplan-Meier plots showing the survival probability of primary ccRCC patients with high and low expression levels of mCAF_2 signature genes. HR, hazard ratio. Red line indicates patients expressed higher mCAF_2 signature genes, grey line indicates patients expressed lower mCAF_2 signature genes. *P* value was calculated by log-rank test. HR, hazard ratio.

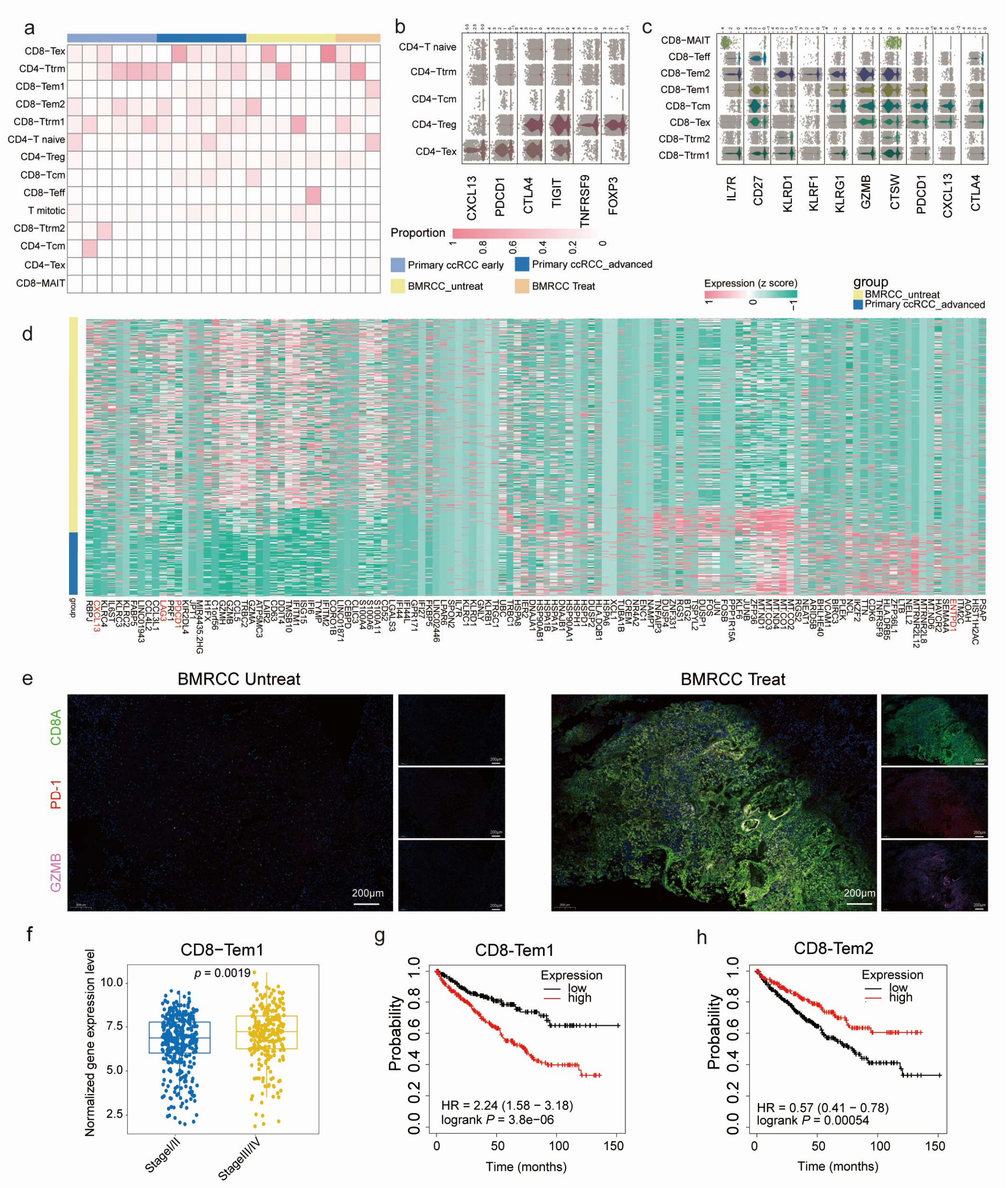

Supplementary Figure 5. Gene expression profiles of T cell subsets in the primary ccRCC and BMRCC.

a. Heatmap showing the fractions of $CD4^+$ and $CD8^+$ T cell subtypes in each sample included in the study. Color from pink to white indicates a high to low cell proportion. b. Violin plot showing the expression levels of Treg marker and exhausted marker genes across $CD4^+$ T cell subtypes. Colors on the columns indicate celltypes marked by y-axis. c. Violin plot showing the expression levels of exhausted marker and effect marker genes across $CD8^+$ T cell clusters. Colors on the columns indicate celltypes marked by y-axis. d. Heatmap showing the differentially expressed genes between primary ccRCCs of advanced stages and treatment-naïve BMRCCs. The single cells are ordered by their tissue origins marked by the legend column on the left. Color from pink to green indicates a high to low gene expression. e. Immunostaining

experiments showing the existence of CD8-Tem1 in treatment-naïve and treated BMRCCs. The green color indicates the expression of CD8 protein. The red color indicates the expression of PD-1 protein. The green color indicates the expression of CD47 protein. The purple color indicates the expression of GZMB protein. f. Comparison of CD8-Tem1 marker genes expression between the early (Stage I/II) and late (Stage III/IV) staged primary ccRCCs using the TCGA-KIRC RNA-seq data. Blue columns indicate stage I/II samples, yellow columns indicate stage III/IV samples. Centre line indicates median, box represents first and third quantiles, and whiskers indicate maximum and minimum values. *P* values were calculated by Student's t test, n= 606 biologically independent samples. Effect size of Cohen's d: 0.261. g, h. Kaplan-Meier plots showing the survival probability of primary ccRCC patients with high and low expression levels of CD8-Tem1 g) or CD8-Tem2 h) signature gene sets. Red line indicates patients expressed higher CD8-Tem1/CD8-Tem2 signature genes CD8-Tem1 g) or CD8-Tem2 h) signature genes expression, grey line indicates patients expressed lower CD8-Tem1 g) or CD8-Tem2 h) signature genes expression. *P* values were calculated by log-rank test. HR, hazard ratio.

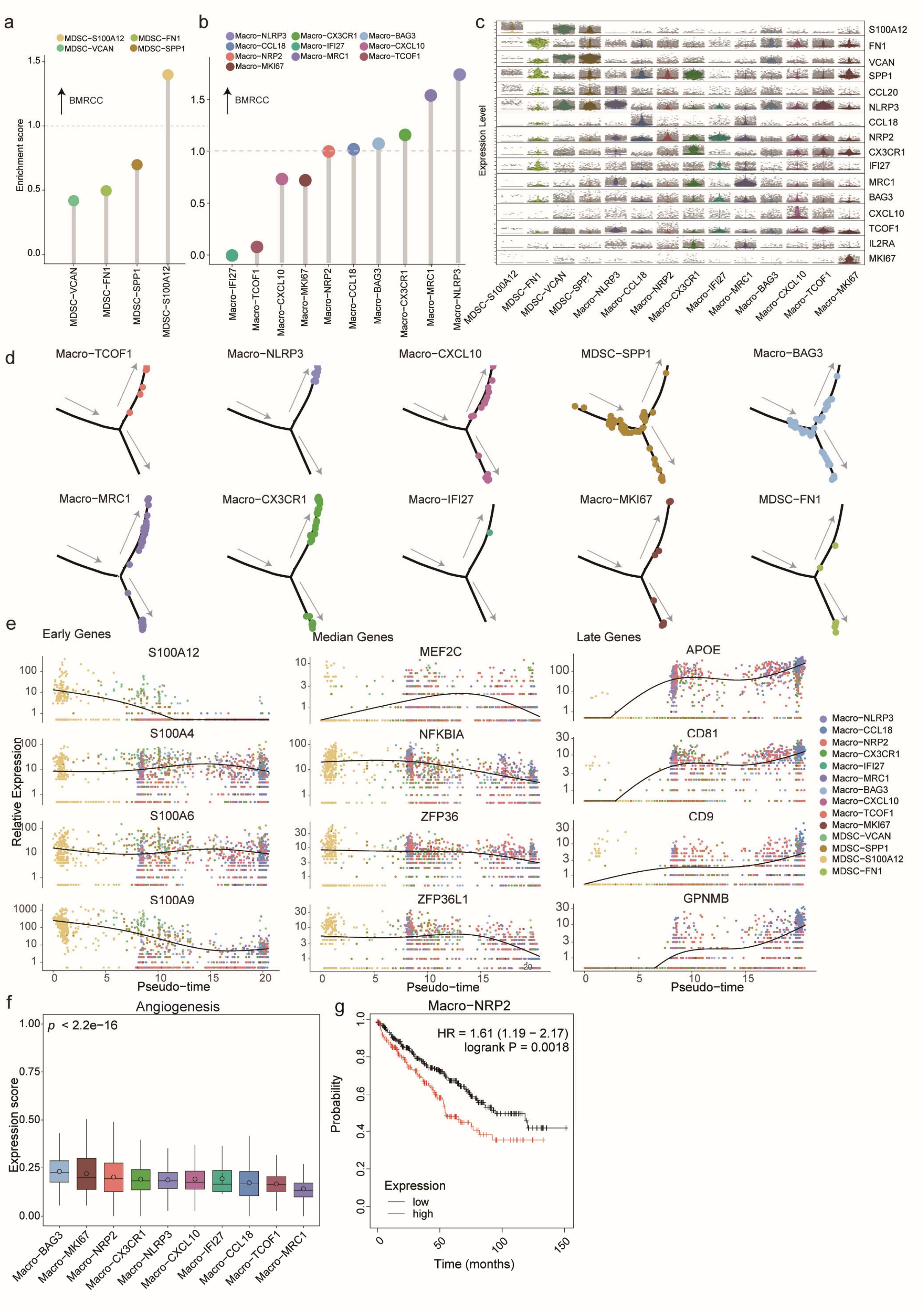

Supplementary Figure 6. The characteristics of myeloid cells in the primary ccRCC and BMRCC.

a. Lollipop plot showing the tissue distribution of MDSC subtypes by Ro/e analysis in BMRCCs in comparison with primary ccRCCs. Dot colour indicate the cell type labelled on x-axis. BMRCC-enriched types were characterized with Ro/e > 1. b. Lollipop plot showing the tissue distribution of macrophage subtypes by Ro/e analysis

in BMRCCs in comparison with primary ccRCCs. Dot colour indicate the cell type labelled on x-axis. BMRCC-enriched cell types were characterized with Ro/e > 1. c. Violin plot showing the marker genes of MDSC and macrophages subtypes. Colors on the columns indicate cell type marked on x-axis. d. Developmental trajectory of MDSC and macrophage subsets, which dominant in BMRCC. Colours of cell indicate cell type marked by legend. e. The patterns of gene expression and cell density along with the pseudotime. Dot colors represent cell subtypes. Colours of cell indicate cell type marked by legend. f. The expression levels of pro-angiogenesis gene signatures among the macrophage subtypes, Colours of cell indicate cell type marked by legend. Centre line indicates median, box represents first and third quantiles, and whiskers indicate maximum and minimum values. *P* values were calculated by Kruskal−Wallis test, n= 5172 biologically independent cells. g. Kaplan-Meier plots showing the survival probability of primary ccRCC patients with high and low expression levels of Macro-NRP2 signature genes. Red line indicates patients expressed higher Macro-NRP2 signature genes expression, grey line indicates patients expressed lower Macro-NRP2 signature genes expression. *P* value was calculated by log-rank test. HR, hazard ratio.

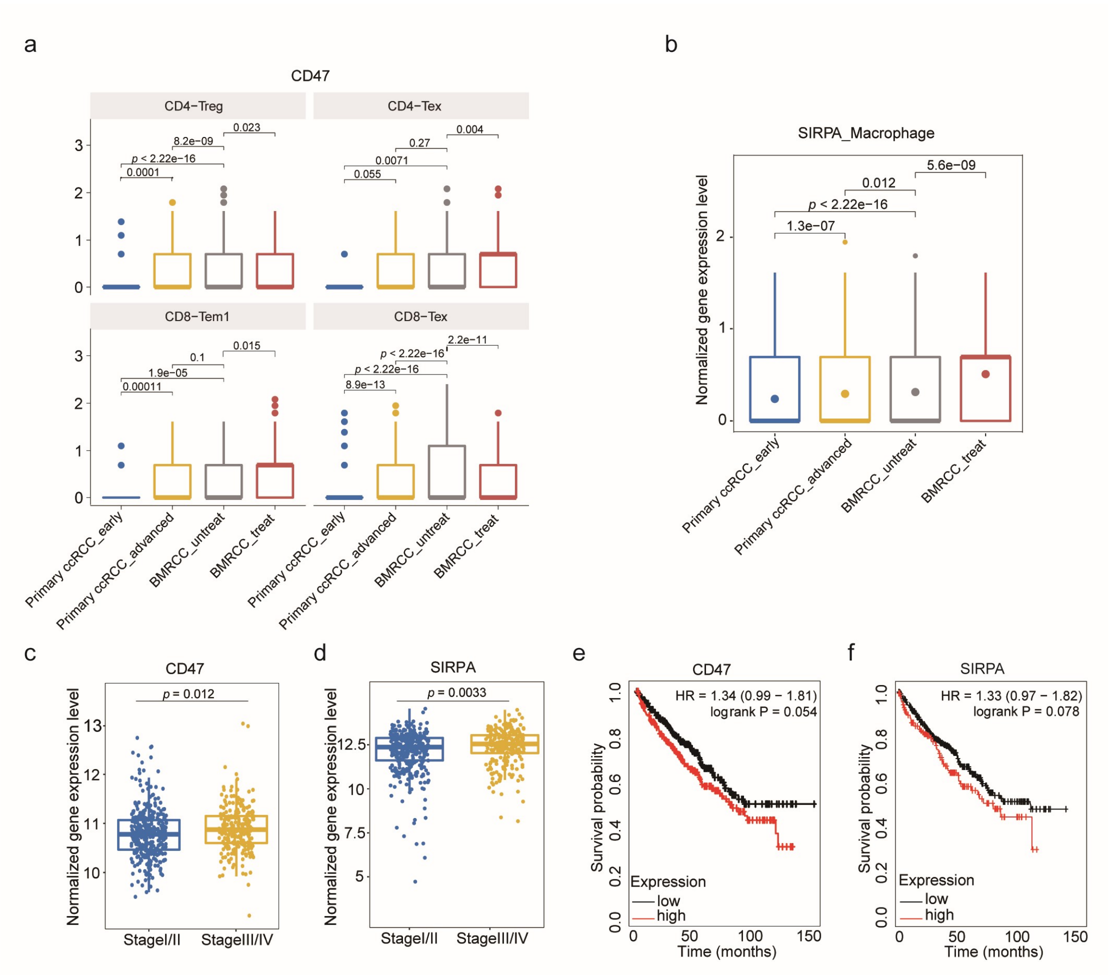

Supplementary Figure 7. Cell-cell interactions in the tumor microenvironment of ccRCC.

a. Comparison of *CD47* expression levels in the indicated T cell subtypes among primary ccRCCs of early and advanced stages and BMRCCs with or without treatment. The blue columns indicate primary ccRCC_early group. The yellow columns indicate primary ccRCC_advanced group. The grey columns indicate BMRCC_Untreat group. The red columns indicate BMRCC_Treat group. *P* values were calculated by wilcox rank sum test. CD4-Treg: n= 3184 biologically independent cells, Effect sizes of Cohen's d for BMRCC_Treat vs BMRCC_Untreat, primary ccRCC early vs BMRCC_Untreat, primary ccRCC_advanced vs BMRCC_Untreat, primary ccRCC_early vs primary ccRCC_advanced are 0.120, 0.485, 0.278 and 0.244, separately. CD4-Tex: n= 861 biologically independent cells, Effect size of Cohen's d: 0.177, 0.659, 0.208 and 0.587, separately. CD8-Tem1: n= 4187 biologically independent cells, Effect size of Cohen's d: 0.178, 0.556, 0.165 and 0.412, separately. CD8-Tex: n= 15925 biologically independent cells, Effect size of Cohen's d: 0.323, 0.498, 0.294 and 0.255, separately. b. Comparison of *SIRPA* expression levels in the macrophages among primary ccRCCs of early and advanced stages and BMRCCs with or without treatment. The blue columns indicate primary

ccRCC_early group. The yellow columns indicate primary ccRCC_advanced group. The grey columns indicate BMRCC_Untreat group. The red columns indicate BMRCC_Treat group. *P* values were calculated by wilcox rank sum test, n= 11481 biologically independent cells. Effect sizes of Cohen's d for BMRCC_Treat vs BMRCC_Untreat, primary ccRCC early vs BMRCC_Untreat, primary ccRCC_advanced vs BMRCC_Untreat, primary ccRCC_early vs primary ccRCC_advanced are 0.493, 0.195, 0.048 and 0.145, separately. c, d. Comparison of *CD47* c) and *SIRPA* d) expression levels between the early (Stage I/II) and late (Stage III/IV) staged primary ccRCCs. *P* values were calculated by wilcox rank sum test, n= 606 biologically independent samples. Effect sizes of Cohen's d for *CD47* and *SIRPA* are 0.206 and 0.236, separately. e, f. Kaplan-Meier plots showing the survival probability of primary ccRCC patients with high and low expression levels of *CD47* e) or *SIRPA* f). Red line indicates patients expressed higher *CD47* e) or *SIRPA* f) expression, grey line indicates patients expressed lower *CD47* e) or *SIRPA* f) expression. *P* values were calculated by log-rank test. HR, hazard ratio. Box-and-whisker plots (a-d): Centre line indicates median, box represents first and third quantiles, and whiskers indicate maximum and minimum values.

Supplementary Table 1: The clinical information of patients.

Samples	Types	Additional treatments	Age	Gender	Other distant metastasis	Surgical site	TNM stages
P01_P	Primary lesion	None	59	Female	None	Right kidney	Stage I
P02_P	Primary lesion	None	66	Male	None	Right kidney	Stage I
P03_P	Primary lesion	None	62	Male	None	Left kidney	Stage III
P04_P	Primary lesion	None	47	Male	None	Left kidney	Stage I
P05_P	Primary lesion	None	67	Female	None	Right kidney	Stage I
P06_P	Primary lesion	None	61	Male	None	Left kidney	Stage I
P07_M	Bone metastasis lesion	Pazopanib, Pebolizumab	47	Male	Yes (chest wall)	T7	Stage IV
P08_M	Bone metastasis lesion	None	67	Male	None	L5	Stage IV
P09_M	Bone metastasis lesion	None	43	Male	Yes (thoracic vertebrae)	The 4th left rib	Stage IV
P10_M	Bone metastasis lesion	None	63	Male	None	T10-11	Stage IV
P11_M1	Bone metastasis lesion	None	68	Male	None	Left femur	Stage IV
P11_M2	Bone metastasis lesion	None	68	Male	None	L4	Stage IV
P12_M	Bone metastasis lesion	None	54	Male	Yes (retroperitoneal lymph node)	T10	Stage IV
P13_M	Bone metastasis lesion	Axitinib, PD-1 inhibitor	77	Male	Left triceps surae	sacrum	Stage IV
P14_M	Bone metastasis lesion	PD-1 inhibitor, Farmitinib	53	Male	None	L3	Stage IV

T: thoracic vertebrae; L: lumbar vertebra; The TNM stages were evaluated according to the TNM classification of the American Joint Committee on Cancer (AJCC cancer staging manual, seventh edition).

Supplementary Table 2: The scRNA data information of samples.

Samples	Cell number	Mean Reads	Median Genes	Q30 Barcode	Q30 RNA Read
P01_P	5054	98366	2930	0.952	0.892
P02_P	8517	36199	1519	0.956	0.892
P03_P	4899	55917	1394	0.964	0.866
P04_P	4777	61439	1880	0.969	0.934
P05_P	4545	56826	1261	0.955	0.892
P06_P	4202	61029	1356	0.964	0.925
P07_M	9423	60295	1173	0.958	0.918
P08_M	10577	69812	1185	0.956	0.914
P09_M	5576	49794	2073	0.958	0.907
P10_M	7747	67646	1872	0.958	0.926
P11_M1	5113	36542	1672	0.966	0.913
P11_M2	6731	56169	911	0.96	0.924
P12_M	7235	58905	1664	0.962	0.93
P13_M	8494	45281	1335	0.956	0.89
P14_M	3686	82215	2239	0.959	0.908

Supplementary Table 3. Signature Genes Used to Define M1, M2, Angiogenesis and Phagocytosis Phenotypes.

M1	M2	Angiogenesis	Phagocytosis
IL23A	IL4R	CCNE1	MRC1
TNF	CCL13	CD44	CD163
CXCL9	CCL20	CXCR4	MERTK
CXCL10	CCL17	E2F3	C1QB
CXCL11	CCL18	EDN1	
CD86	CCL22	EZH2	
IL1A	CCL24	FGF18	
IL1B	LYVE1	FGFR1	
IL6	VEGFA	FYN	
CCL5	VEGFB	HEY1	
IRF5	VEGFC	ITGAV	
IRF1	VEGFD	JAG1	
CD40	EGF	JAG2	
IDO1	CTSA	MMP9	
KYNU	TGFB1	NOTCH1	
CCR7	TGFB2	PDGFA	
	TGFB3	PTK2	
	MMP14	SPP1	
	MMP19	STC1	
	MMP9	TNFAIP6	
	CLEC7A	TYMP	
	FASLG	VAV2	
	TNFSF12	VCAN	
	TNFSF8	VEGFA	
	CD276		
	MSR1		
	FN1		
	IRF4		
