# Supplementary material for: Single-cell profiling of the microenvironment in human bone metastatic renal cell carcinoma
Source: Commun Biol. 2024 Jan 12;7:91. doi: 10.1038/s42003-024-05772-y (PMC10786927; doi:10.1038/s42003-024-05772-y)
Supplement: Supplementary file 2 — Description of Additional Supplementary Files [file 42003_2024_5772_MOESM2_ESM.pdf]

Description of Additional Supplementary Files

File name: Supplementary Data 1
Description: Marker genes of myeloid and T cells subtypes.

File name: Supplementary Data 2
Description: Source data of Fig 1c-f, 2b-e, 3ce, 4b, 4d, 4f, 4j, 5c, 5f, 6c-e, 7a, 7c-e, 7g.
