# Supplementary material for: Single-cell profiling of the microenvironment in human bone metastatic renal cell carcinoma
Source: Commun Biol. 2024 Jan 12;7:91. doi: 10.1038/s42003-024-05772-y (PMC10786927; doi:10.1038/s42003-024-05772-y)
Supplement: Supplementary file 5 — Reporting Summary [file 42003_2024_5772_MOESM5_ESM.pdf]

nature portfolio

Corresponding author(s): Wangjun Yan

Last updated by author(s): Dec 8, 2023

Reporting Summary

Nature Portfolio wishes to improve the reproducibility of the work that we publish. This form provides structure for consistency and transparency in reporting. For further information on Nature Portfolio policies, see our Editorial Policies and the Editorial Policy Checklist.

Statistics

For all statistical analyses, confirm that the following items are present in the figure legend, table legend, main text, or Methods section.

n/a	Confirmed	
☐	☒	The exact sample size (*n*) for each experimental group/condition, given as a discrete number and unit of measurement
☐	☒	A statement on whether measurements were taken from distinct samples or whether the same sample was measured repeatedly
☐	☒	The statistical test(s) used AND whether they are one- or two-sided<br>*Only common tests should be described solely by name; describe more complex techniques in the Methods section.*
☒	☐	A description of all covariates tested
☐	☒	A description of any assumptions or corrections, such as tests of normality and adjustment for multiple comparisons
☐	☒	A full description of the statistical parameters including central tendency (e.g. means) or other basic estimates (e.g. regression coefficient) AND variation (e.g. standard deviation) or associated estimates of uncertainty (e.g. confidence intervals)
☐	☒	For null hypothesis testing, the test statistic (e.g. *F*, *t*, *r*) with confidence intervals, effect sizes, degrees of freedom and *P* value noted<br>*Give P values as exact values whenever suitable.*
☒	☐	For Bayesian analysis, information on the choice of priors and Markov chain Monte Carlo settings
☒	☐	For hierarchical and complex designs, identification of the appropriate level for tests and full reporting of outcomes
☐	☒	Estimates of effect sizes (e.g. Cohen's *d*, Pearson's *r*), indicating how they were calculated

Our web collection on statistics for biologists contains articles on many of the points above.

Software and code

Policy information about availability of computer code

Data collection	Single cell RNA sequencing data was generated from the 10× Genomics platform and sequenced on Illumina NovaSeq 6000 Systems.
Data analysis	CellRanger v6.0.2; Seurat v4.0.5

For manuscripts utilizing custom algorithms or software that are central to the research but not yet described in published literature, software must be made available to editors and reviewers. We strongly encourage code deposition in a community repository (e.g. GitHub). See the Nature Portfolio guidelines for submitting code & software for further information.

Data

Policy information about availability of data

All manuscripts must include a data availability statement. This statement should provide the following information, where applicable:

- Accession codes, unique identifiers, or web links for publicly available datasets
- A description of any restrictions on data availability
- For clinical datasets or third party data, please ensure that the statement adheres to our policy

All the sequencing data were deposited in the National Omics Data Encyclopedia (NODE) under accession number OEP004678 (https://www.biosino.org/node/project/detail/OEP004678).

Research involving human participants, their data, or biological material

Policy information about studies with human participants or human data. See also policy information about sex, gender (identity/presentation), and sexual orientation and race, ethnicity and racism.

Reporting on sex and gender	Patients of samples including 2 female and 12 male
Reporting on race, ethnicity, or other socially relevant groupings	N/A.
Population characteristics	3 patients were treated with tyrosine kinase inhibitor (TKI) and PD-1 inhibitor, others were treated naive.
Recruitment	Patients who were pathologically diagnosed with clear cell renal cell carcinoma, were enrolled in this study
Ethics oversight	This study was reviewed and approved by the Institutional Review Board of Fudan University Shanghai Cancer Center.

Note that full information on the approval of the study protocol must also be provided in the manuscript.

Field-specific reporting

Please select the one below that is the best fit for your research. If you are not sure, read the appropriate sections before making your selection.

☒ Life sciences ☐ Behavioural & social sciences ☐ Ecological, evolutionary & environmental sciences

For a reference copy of the document with all sections, see nature.com/documents/nr-reporting-summary-flat.pdf

Life sciences study design

All studies must disclose on these points even when the disclosure is negative.

Sample size	A sample size for the current experimental study was limited to an original clinical trial. In total, ~250,000 cells from 14 patients were sequenced.
Data exclusions	Seqeuencing data were processed and filtered using a well-established pipeline, and only single cells passing quality control were retained for further analyses
Replication	All attempts at replication were successful, n is described on each figure.
Randomization	The samples were collected randomly
Blinding	Blinding was not necessary during data collection, because we worked with 14 donors that had RCC or BMRCC.

Reporting for specific materials, systems and methods

We require information from authors about some types of materials, experimental systems and methods used in many studies. Here, indicate whether each material, system or method listed is relevant to your study. If you are not sure if a list item applies to your research, read the appropriate section before selecting a response.

Materials & experimental systems

n/a	Involved in the study	
☐	☒	Antibodies
☒	☐	Eukaryotic cell lines
☒	☐	Palaeontology and archaeology
☒	☐	Animals and other organisms
☒	☐	Clinical data
☒	☐	Dual use research of concern
☒	☐	Plants

Methods

n/a	Involved in the study	
☒	☐	ChIP-seq
☒	☐	Flow cytometry
☒	☐	MRI-based neuroimaging

Antibodies

Antibodies used	FAP(abcam, ab218164, Rabbit pAb, 1:1000); Vimentin (CST, 5741, Rabbit mAb, 1:1000); CD8A (abclonal, A0663, Rabbit mAb, 1:1000); PD-1 (abclonal, A20217, Mouse mAb, 1:1000); GZMB (abclonal, A22993, Rabbit mAb, 1:1000); CD68 (abclonal, A23205, Rabbit mAb,

1:1000); NRP2 (proteintech, 11268-1-AP, Rabbit pAb, 1:1000); SPP1(proteintech, 22952-1-AP, Rabbit pAb, 1:1000); CD47 (SCBT, sc-12730, Mouse mAb, 1:500); SIRPA (abcam, ab260039, Rabbit mAb, 1:1000)

Validation

FAP(abcam, ab218164, Rabbit polyclonal): Specificity were comfirmed in this study and other studies (PMID: 34479922, PMID: 32855207) by manufacturer (https://www.abcam.cn/products/primary-antibodies/fibroblast-activation-protein-alpha-antibody-ab218164.html)
Vimentin (CST, 5741, Rabbit mAb): Specificity were comfirmed in this study and other studies (PMID: 37322019, PMID: 37291192, PMID: 37237004) by manufacturer (https://www.cellsignal.cn/products/primary-antibodies/vimentin-d21h3-xp-rabbit-mab/5741)
CD8A (abclonal, A0663, Rabbit mAb): Specificity were comfirmed in this study and other studies (PMID: 35640059, PMID: 35493068) by manufacturer (https://abclonal.com.cn/catalog/A0663)
PD-1 (abclonal, A20217, Mouse mAb): Specificity were comfirmed in this study by manufacturer (https://abclonal.com.cn/catalog/A20217)
GZMB (abclonal, A22993, Rabbit mAb): Specificity were comfirmed in this study by manufacturer (https://abclonal.com.cn/catalog/A22993)
CD68 (abclonal, A23205, Rabbit mAb): Specificity were comfirmed in this study by manufacturer (https://abclonal.com.cn/catalog/A23205)
NRP2 (proteintech, 11268-1-AP, Rabbit pAb): Specificity were comfirmed in this study and other studies (PMID: 29270148, PMID: 30599272, PMID: 37095084) by manufacturer (https://www.ptgcn.com/products/NELL2-Antibody-11268-1-AP.htm)
SPP1(proteintech, 22952-1-AP, Rabbit pAb): Specificity were comfirmed in this study and other studies (PMID: 35245443, PMID: 31915373, PMID: 25368378) by manufacturer (https://www.ptgcn.com/products/OPN,-SPP1-Antibody-22952-1-AP.htm)
CD47 (SCBT, sc-12730, Mouse mAb): Specificity were comfirmed in this study and other studies (PMID: 36939440, PMID: 36898577, PMID: 36823443) by manufacturer (https://www.scbt.com/zh/p/cd47-antibody-b6h12)
SIRPA (abcam, ab260039, Rabbit mAb): Specificity were comfirmed in this study and other studies (PMID: 35317832, PMID: 36158683) by manufacturer (https://www.abcam.cn/products/primary-antibodies/sirp-alpha-antibody-epr22930-163-ab260039.html)

Plants

Seed stocks

N/A.

Novel plant genotypes

N/A.

Authentication

N/A.
